# Population-Level Health Benefits and Harms Associated With Buprenorphine/Naloxone vs Methadone

**DOI:** 10.1001/jamanetworkopen.2025.51337

**Published:** 2025-12-26

**Authors:** Benjamin Enns, Brenda Carolina Guerra-Alejos, Jeong Eun Min, Addie Carter, Uwe Siebert, Bohdan Nosyk

**Affiliations:** 1 Centre for Advancing Health Outcomes, Vancouver, British Columbia, Canada; 2 Faculty of Health Sciences, Simon Fraser University, Burnaby, British Columbia, Canada; 3 Center for Health Decision Science, Harvard University T.H. Chan School of Public Health, Boston, Massachusetts; 4 Department of Epidemiology, Harvard University T.H. Chan School of Public Health, Boston, Massachusetts; 5 Department of Health Policy and Management, Harvard University T.H. Chan School of Public Health, Boston, Massachusetts; 6 Department of Public Health, Health Services Research and Health Technology Assessment, UMIT TIROL—University for Health Sciences and Technology, Tirol, Austria; 7 Institute for Technology Assessment, Massachusetts General Hospital, Harvard Medical School, Boston; 8 Department of Radiology, Massachusetts General Hospital, Harvard Medical School, Boston

## Abstract

**Importance:**

In 2017, British Columbia guidelines changed the preferred first-line treatment for opioid use disorder from methadone to buprenorphine/naloxone. Systematic reviews have consistently shown better treatment retention associated with methadone, while studies have reported a reduced risk of mortality associated with buprenorphine/naloxone, causing confusion in interpretation for clinical guidelines.

**Objective:**

To estimate the population-level health benefits and harms associated with buprenorphine/naloxone vs methadone for treatment of opioid use disorder, accounting for differences in treatment retention and risk of mortality while receiving treatment.

**Design, setting, and participants:**

This decision analytic model analysis was conducted from January 1, 2010, to March 17, 2020, using a semi-Markov cohort model and linked population-level health administrative data on all individuals presenting for opioid agonist treatment in British Columbia, Canada. Hazard ratios were estimated for the association of buprenorphine/naloxone vs methadone with treatment discontinuation (breaks in dispensations lasting ≥5 days for methadone and ≥6 days for buprenorphine/naloxone) and mortality while receiving treatment (per protocol). Treatment episodes in primary analysis were based on the medication initiated (initiator analysis). Probabilistic (mean estimates and 95% credible intervals from 10 000 simulations) and deterministic sensitivity analyses were conducted. Data were analyzed between August 2023 and October 2024.

**Exposure:**

Alternative treatment policies in which buprenorphine/naloxone or methadone were exclusively available to individuals presenting for opioid agonist treatment.

**Main outcome and measures:**

Incremental life-years, fatal overdoses, and all-cause deaths.

**Results:**

The study population included 40 461 individuals with 109 126 cumulative person-years of follow-up (median [IQR] age, 33 [23-43] years; 66.0% [range, 56.0%-76.0%] male). A policy of exclusively buprenorphine/naloxone was estimated to have −1602 incremental life-years (95% credible interval, −3249 to −549 life-years) compared with methadone, with an additional 221 fatal overdoses (95% credible interval, 119 to 376 overdoses) and 303 all-cause deaths (95% credible interval, 120 to 589 deaths) over a 10-year period.

**Conclusions and relevance:**

Results from this study suggest that any advantages from a reduced risk of mortality with treatment with buprenorphine/naloxone were outweighed by deficits in treatment retention. Evaluated in a jurisdiction where both medications were available in office-based settings, these findings do not support recommendations of buprenorphine/naloxone as first-line treatment over methadone.

## Key Points

**Question:**

What are the combined population-level health outcomes associated with treatment with buprenorphine/naloxone compared with methadone, accounting for differences in treatment retention and risk of mortality?

**Findings:**

This decision analytical modeling study among 40 461 individuals receiving opioid agonist treatment found population health losses with buprenorphine/naloxone compared with methadone across all model specifications, subgroups, and sensitivity analyses. Any potential advantages from a reduced risk of mortality while individuals were receiving treatment with buprenorphine/naloxone were outweighed by deficits in treatment retention.

**Meaning:**

Findings from this study do not support recommendations of buprenorphine/naloxone as first-line treatment over methadone.

## Teaser

This decision analytical model estimates the incremental life-years, fatal overdoses, and all-cause deaths among individuals with opioid use disorder treated with buprenorphine/naloxone vs methadone.

## Introduction

Methadone and buprenorphine/naloxone are the 2 most common medications for treatment of opioid use disorder (OUD).^1,2^ In Canada, methadone has been used as an opioid agonist treatment (OAT) for OUD since 1959,^3^ with buprenorphine/naloxone approved in 2007.^4^ In 2017, 14 months after the drug overdose public health emergency was declared in British Columbia (BC),^5^ clinical guidelines for OUD were published in which BC changed its preferred first-line treatment from methadone to buprenorphine/naloxone.^6^ Canadian guidelines from the Canadian Research Initiative in Substance Matters^7^ and Centre for Addiction and Mental Health^8^ followed suit shortly thereafter. In Canada, both buprenorphine/naloxone and methadone are available in office-based settings and specialized drug treatment centers, covered under a universal health care system. In the US, opioid treatment programs can prescribe methadone and buprenorphine/naloxone; however, methadone is classified as having higher potential for dependency and abuse than buprenorphine/naloxone and is thus prohibited in office-based settings.^9,10^

Systematic reviews have consistently shown better treatment retention associated with methadone,^11,12,13^ while retrospective cohort studies have reported a lower risk of mortality associated with buprenorphine/naloxone while receiving treatment.^14,15^ However, empirical evidence on the comparative effectiveness of treatment options is limited by short follow-up periods,^16,17^ selection criteria that limit generalizability,^16,17,18^ and the rapidly changing unregulated drug supply.^19,20^ A 2024 retrospective cohort study using population-level data^21^ found that methadone was superior to buprenorphine/naloxone in promoting treatment retention, with an adjusted hazard ratio (HR) of 1.58 (95% CI, 1.53-1.63) for clients with incident OAT and 1.44 (95% CI, 1.41-1.47) for clients with prevalent new OAT. Although results on mortality favored buprenorphine/naloxone (incident OAT: HR, 0.57; 95% CI, 0.24-1.35; prevalent new OAT: HR, 0.97; 95% CI, 0.54-1.73), the risk of mortality was low for both treatments; therefore, measures of association were estimated with a high degree of uncertainty.

Estimating the combined outcomes that are associated with discordant comparative effectiveness results requires a decision-analytic model that captures the cumulative time individuals spend in and out of treatment while accounting for the changing risk of overdose over time. The US Preventive Services Task Force (USPSTF) has established that decision models are warranted when there are outstanding clinical questions for which a systematic review is unlikely to determine net benefits.^22^ The USPSTF previously used modeling studies to make recommendations^23^ on screening and preventive medications for chronic diseases, such as cardiovascular disease,^24^ and for lung,^25^ breast,^26^ cervical,^27^ and colorectal cancer.^28^ In the context of OAT, there is limited and conflicting population-level evidence on the net effects of treatment alternatives to inform clinical recommendations, particularly in the fentanyl era.^11^ Our objective was to estimate the net population-level benefits and harms associated with buprenorphine/naloxone vs methadone for treatment of OUD in BC, Canada, comparing outcomes from 2 alternative treatment policies using a decision-analytic model.

## Methods

This decision analytical model is reported following the Consolidated Health Economic Evaluation Reporting Standards (CHEERS) reporting guideline. Providence Health Care Research Institute and the Simon Fraser University Office of Research Ethics determined that this study was exempt from research ethics board review and informed consent per article 2.5 of the Tri-Council Policy Statement: Ethical Conduct for Research Involving Humans.

### Alternative Treatment Policy Analysis

We conducted our analysis from January 1, 2010, to March 17, 2020, comparing outcomes in 2 counterfactual treatment policies: (1) buprenorphine/naloxone exclusively available for individuals seeking pharmacological treatment for opioid use disorder and (2) methadone exclusively available for individuals seeking pharmacological treatment for OUD (Table 1).^14,21^ HRs on treatment discontinuation (breaks in dispensations lasting ≥5 days for methadone and ≥6 days for buprenorphine/naloxone) and mortality while receiving treatment based on per-protocol analysis were taken from a study designed to emulate a target trial using observational data.^21^ This design was used to address confounding by indication at baseline, time-varying confounding due to suboptimal dosing, and the effects of take-home dosing for individuals initiating buprenorphine/naloxone or methadone by adjusting for treatment setting, baseline client characteristics, and treatment characteristics. We conducted our primary analysis using an initiator specification, in which treatment episodes were defined according to the medication initiated and censored only at death or the end of follow-up. Individuals in initiator analysis could switch treatment medications within episodes, although this occurred in less than 5% of all episodes (4807 of 97 516 episodes [4.9%]). We assumed that individuals would start OAT with either treatment if offered; that is, we did not differentiate by treatment type for individuals initiating or returning to OAT and estimated these probabilities for OAT reinitiation with either medication. Apart from these differences, study populations for counterfactual groups, representing all individuals accessing OAT in BC during the study period, were identical. We scaled model outcomes by the number of individuals accessing OAT in each year (eTable 1 in Supplement 1). HRs were reestimated to capture outcomes for individuals with prior OAT experience and thus differ slightly from the prevalent new user definition (eAppendix 1 in Supplement 1).

**Table 1.** Alternative Treatment Policies and Integration of Comparative Effectiveness Estimates

Alternative treatment policy	Modeling relative differences in comparative effectiveness	
	Treatment retention	Mortality in treatment
Status quo (observed): all individuals initiating or reinitiating OAT between January 2010 and March 2020 in BC accessed OAT treatment as observed in population-level data.	Weekly time-dependent retention probabilities derived from Weibull hazard function for treatment discontinuation, estimated from population-level data for individuals receiving OAT (combined).	Rate parameters used to calculate overdose and mortality probabilities calibrated to target data for individuals in and out of OAT, derived from population-level data.
Methadone exclusively available (counterfactual): all individuals initiating or reinitiating OAT between January 2010 and March 2020 in BC received exclusively methadone.	Weekly time-dependent retention probabilities derived from Weibull hazard function for treatment discontinuation, estimated from population-level data for individuals receiving methadone.	Rates of fatal overdose and nonoverdose mortality in treatment estimated via model calibration used for methadone.
BNX exclusively available (counterfactual): all individuals initiating or reinitiating OAT between January 2010 and March 2020 in BC received exclusively BNX.	Weekly time-dependent retention probabilities derived from HR for treatment discontinuation from Nosyk et al,^21^ 2024, applied to time-dependent hazard function estimated for methadone retention.	HR for BNX on mortality in treatment derived from Nosyk et al,^21^ 2024, multiplied to base rates of fatal overdose (including first week after treatment discontinuation; Pearce et al,^14^ 2020) and nonoverdose mortality used for methadone.

Abbreviations: BC, British Columbia; BNX, buprenorphine/naloxone; HR, hazard ratio; OAT, opioid agonist treatment.

### Statistical Analysis

#### Model Description

We used a decision-analytic state-transition semi-Markov cohort model adapted from a previously validated model used to estimate the cost-effectiveness of OAT treatment in several North American contexts.^29,30,31^ We followed international best practices.^32,33^ and reporting guidelines. The model included 6 mutually exclusive health states: buprenorphine/naloxone, methadone, out of treatment, long-term abstinence, overdose, and death (eFigure 1 in Supplement 1). We incorporated time dependence for each health state, which made the probability of discontinuation from a given health state a function of time spent in that state. We also incorporated changes in the risk of mortality by calendar year; parameters that determined the underlying risk of overdose thus varied annually. In weekly cycles, individuals could remain in their current health state, transition to other health states, or die. We stratified the model population by clients receiving incident OAT (OAT naive) and experienced clients (≥1 prior OAT episode) to capture the estimated difference in HRs and the increasing proportion of experienced clients receiving OAT who were initiating episodes over time.

The probability of overdose varied by health state and accounted for the increased risk of overdose during the first week after treatment discontinuation, as well as the impact of fentanyl in the unregulated drug supply. The risk of fatal overdose was adjusted to account for the probability of naloxone reversal, which increased rapidly from 2016 as BC scaled up its take-home naloxone program.^34^ Individuals experiencing a nonfatal overdose could transition to treatment, remain out of treatment, or overdose again the following week.

Our primary outcome was the cumulative difference in incremental life-years between alternative treatment policies, and we also reported differences in fatal overdoses and all-cause deaths. Mean estimates and 95% credible intervals (CrIs) of 10 000 probabilistic simulations were estimated for each outcome, where parameter sets were drawn randomly from distributions specified by parameter type (Table 2; eTable 2 in Supplement 1).^19,21,35,36,37,38,39,40^ Finally, we reported the percentage of simulations that had net health gains or losses for alternative treatment policies.

**Table 2.** Estimates for Model Parameters

Parameter	Estimate (range)		Source	Distribution
	Initiator analysis	Per-protocol analysis		
Population characteristics at baseline				
Cohort starting age, median (IQR), y	33 (23-43)	33 (23-43)	Population-level data	NS
Gender mix, % (95% CI)				
Male	66.0 (56.0-76.0)	66.0 (56.0-76.0)	Population-level data	NS
Female	34.0 (24.0-44.0)	34.0 (24.0-44.0)		
Overdose				
Rates of overdose and fatal overdose				
Nonfentanyl overdose rate, OAT (95% CrI)	0.0012 (0.0011-0.0012)	0.0010 (0.0009-0.0011)	Calibrated^a^	Posterior
Overdose: RR for out of treatment vs OAT (95% CI)	2.05 (1.77-2.33)	2.05 (1.77-2.33)	Population-level data	Log normal
Fatal overdose rate, OAT (95% CrI)	0.0071 (0.0066-0.0076)	0.0070 (0.0063-0.0078)	Calibrated^a^	Posterior
Fatal overdose: RR for out of treatment vs OAT (95% CI)	7.81 (5.79-9.83)	7.81 (5.79-9.83)	Population-level data	Log normal
BNX mortality: fatal overdose: HR for BNX vs methadone (95% CI)				
Incident user	0.57 (0.24-1.35)	0.57 (0.24-1.35)	Nosyk et al,^21^ 2024	Log normal
Incident user, high-dose SA	NA	0.08 (0.01-0.47)	Nosyk et al,^21^ 2024	Log normal
Experienced user	1.32 (0.62-2.78)	1.32 (0.62-2.78)	Nosyk et al,^21^ 2024	Log normal
Experienced user, high-dose SA	NA	1.33 (0.52-3.39)	Nosyk et al,^21^ 2024	Log normal
First-week overdose risk multiplier (95% CI)				
Overdose: RR for first week out of treatment vs week 2 or later	4.04 (1.43-11.43)	4.04 (1.43-11.43)	Durand et al,^35^ 2022	Log normal
Fentanyl and naloxone				
Natural log overdose rate multiplier (fentanyl prevalence)^b^	1.7 (1.61-1.78)	1.72 (1.62-1.82)	Calibrated^a^	Posterior
Natural log overdose rate multiplier (fentanyl delta)^b^	0.69 (0.49-0.89)	0.66 (0.43-0.88)	Calibrated^a^	Posterior
Fentanyl prevalence	eTable 9 in Supplement 1		BC coroner data^19^	NS
Probability of naloxone reversal	eTable 9 in Supplement 1		Irvine et al,^36^ 2019; Lei et al^37^ 2022	NS
Probability of remaining in health state (95% CI)				
Methadone treatment retention (baseline hazard of discontinuation)				
Weibull shape parameter (incident OAT client)	0.496 (0.49-0.503)	0.654 (0.64-0.668)	Population-level data	Weibull
Weibull scale parameter (incident OAT client)	0.191 (0.199-0.184)	0.171 (0.184-0.159)	Population-level data	Weibull
Weibull shape parameter (experienced OAT client)	0.496 (0.493-0.500)	0.700 (0.692-0.708)	Population-level data	Weibull
Weibull scale parameter (experienced OAT client)	0.267 (0.272-0.261)	0.274 (0.283-0.266)	Population-level data	Weibull
BNX treatment retention vs methadone: HR of treatment discontinuation				
Incident user	1.58 (1.53-1.63)	1.67 (1.58-1.76)	Nosyk et al,^21^ 2024	Log normal
Incident user, high-dose SA	NA	1.72 (1.62-1.82)	Nosyk et al,^21^ 2024	Log normal
Experienced user	1.41 (1.37-1.45)	1.36 (1.31-1.42)	Nosyk et al,^21^ 2024	Log normal
Experienced user, high-dose SA	NA	1.33 (1.27-1.39)	Nosyk et al,^21^ 2024	Log normal
Out of treatment (95% CrI)				
Weibull shape parameter	0.974 (0.902-1.039)	1.600 (1.423-1.817)	Calibrated^a^	Posterior
Weibull scale parameter	0.046 (0.037-0.056)	0.016 (0.009-0.023)	Calibrated^a^	Posterior
Long-term abstinence (95% CrI)				
Weibull shape parameter	0.983 (0.933-1.034)	1.353 (1.265-1.453)	Calibrated^a^	Posterior
Weibull scale parameter	0.028 (0.026-0.031)	0.016 (0.013-0.018)	Calibrated^a^	Posterior
Destination health state transitions^c^				
From overdose, % (95% CI)				
Return to treatment (incident OAT client)	2.6 (2.5-2.8)	1.8 (1.7-2)	Population-level data	Dirichlet
Return to treatment (experienced OAT client)	3.6 (3.4-3.7)	2.8 (2.6-2.9)	Population-level data	Dirichlet
Return to opioid use (incident OAT client)	97.4 (97.2-97.5)	98.2 (98-98.3)	Population-level data	Dirichlet
Return to opioid use (experienced OAT client)	96.4 (96.3-96.6)	97.2 (97.1-97.4)	Population-level data	Dirichlet
From methadone, No./total No. (%)				
Return to opioid use (incident OAT client)	18 494/18 981 (97.4)	(15 104/15 507 (97.4)	Population-level data	Dirichlet
Return to opioid use (experienced OAT client)	47 579/48 087 (98.9)	35 830/36 229 (98.9)	Population-level data	Dirichlet
Long-term abstinence (incident OAT client)	487/18 981 (2.6)	403/15 507 (2.6)	Population-level data	Dirichlet
Long-term abstinence (experienced OAT client)	508/48 087 (1.1)	399/36 229 (1.1)	Population-level data	Dirichlet
From BNX, No./total No. (%)				
Return to opioid use (incident OAT client)	11 656/11 910 (97.9)	9880/10 107 (97.8)	Population-level data	Dirichlet
Return to opioid use (experienced OAT client)	18 390/18 538 (99.2)	15 712/15 845 (99.2)	Population-level data	Dirichlet
Long-term abstinence (incident OAT client)	254/11 910 (2.1)	227/10 107 (2.2)	Population-level data	Dirichlet
Long-term abstinence (experienced OAT client)	148/18 538 (0.8)	133/15 845 (0.8)	Population-level data	Dirichlet
From out of treatment: return to treatment	100	100	Assumption	NS
From long-term abstinence: return to opioid use	100	100	Assumption	NS
Nonoverdose mortality				
Long-term abstinence	Equivalent to general population		Stats Canada^38^	NS
RR for OAT vs general population (95% CrI)	4.11 (3.98-4.25)	3.92 (3.78-4.06)	Calibrated^a^	Posterior
RR for out of treatment vs OAT (95% CI)	2.52 (1.9-3.14)	2.52 (1.9-3.14)	Population-level data	Log normal
BNX mortality vs methadone: HR of nonoverdose mortality (95% CI)				
Incident user	0.57 (0.24-1.35)	0.57 (0.24-1.35)	Nosyk et al,^21^ 2024	Log normal
Experienced user	1.32 (0.62-2.78)	1.32 (0.62-2.78)	Nosyk et al,^21^ 2024	Log normal

Abbreviations: BNX, buprenorphine/naloxone; CrI, credible interval; HR, hazard ratio; NA, not applicable; NS, not sampled (included in 1-way sensitivity analysis); OAT, opioid agonist treatment; PSA, probabilistic sensitivity analysis; RR, rate ratio; SA, sensitivity analysis.

^a^ See eTable 4 in Supplement 1 for prior values of calibrated parameters.

^b^ Modeled as exponential multiplier.

^c^ Transitions applied only to a subset of cohort members who discontinued their current health state, did not overdose, and remained alive.

#### Model Parameter Estimation and Data Sources

The dataset used for this analysis included all BC individuals receiving OAT consisting of buprenorphine/naloxone or methadone between January 1, 2010, and March 17, 2020.^21,41^ We estimated population characteristics, health state transition probabilities, and parameters used to derive the probability of overdose and mortality from this dataset, and data were analyzed between August 2023 and October 2024. We used published literature and provincial surveillance data sources to estimate fentanyl prevalence in the unregulated drug supply,^19^ as well as the probability of overdose reversal via naloxone administration (eTable 3 in Supplement 1).^36,37^ Model inputs are presented in Table 2, and details on parameter estimation and datasets are available in eAppendix 2 in Supplement 1.

#### Status Quo and Model Calibration

We calibrated the model on a status quo in which buprenorphine/naloxone and methadone were both available according to observed practice in BC to match annual target data for fatal overdoses, nonoverdose deaths, estimated nonfatal overdoses, proportion of time spent out of treatment, and proportion of time spent in long-term abstinence from 2012 to 2020 (eAppendix 3 in Supplement 1). We used a bayesian calibration approach with incremental mixture importance sampling,^42^ which has been used in prior applications^31^ and is recommended for calibrating health policy models.^43,44,45^ We conducted all statistical and modeling analyses using R statistical software version 4.3.2 (R Project for Statistical Computing).^46^

#### Sensitivity Analysis

We created 2 alternative scenarios as a sensitivity analysis. First, we parameterized the model using HRs estimated from a per-protocol analysis, which defined treatment status more narrowly, censoring episodes with suboptimal dosing, treatment switching, or tapers and adjusting for the effects of medication carries. Second, while estimates of comparative effectiveness for most subgroups did not differ substantially from the full population results,^21^ buprenorphine/naloxone provided at higher daily doses had a lower risk of mortality compared with higher daily doses of methadone among clients with incident OAT, so we also parameterized the model using HR estimates for this high-dose subgroup.

We conducted a deterministic 2-way sensitivity analysis on HRs for the association of buprenorphine/naloxone vs methadone with treatment discontinuation and mortality, simultaneously varying each factor relative to its base point estimate until the alternative treatment was favored. Our goals were to determine whether changes to treatment retention or mortality risk had greater changes in model outcomes, as well as estimating the magnitude of change in parameter values required for the alternative treatment policy to be favored. Finally, we estimated our main results for only clients with incident OAT or experienced clients and conducted univariate (1-way) sensitivity analyses, varying model parameters individually within empirically derived uncertainty ranges (Table 2).

## Results

Our data included 40 461 individuals with 109 126 cumulative person-years of follow-up (median [IQR] age, 33 [23-43] years; 66.0% [range, 56.0%-76.0%] male). Calibrated model outputs fit well to target data, particularly for fatal overdoses and nonoverdose deaths (full calibration results and diagnostic plots are in eAppendix 4, eTable 4, and eFigures 2-7 in Supplement 1). Modeling overdose risk as a function of fentanyl prevalence and the change in fentanyl prevalence allowed the model to match the rapid increase in fatal overdoses from 2015 to 2017. The relative proportion of clients with incident and experienced OAT in the model population also tracked observed proportions closely (eFigure 8 in Supplement 1).

### Alternative Treatment Policy Analysis

**Figure 1.**
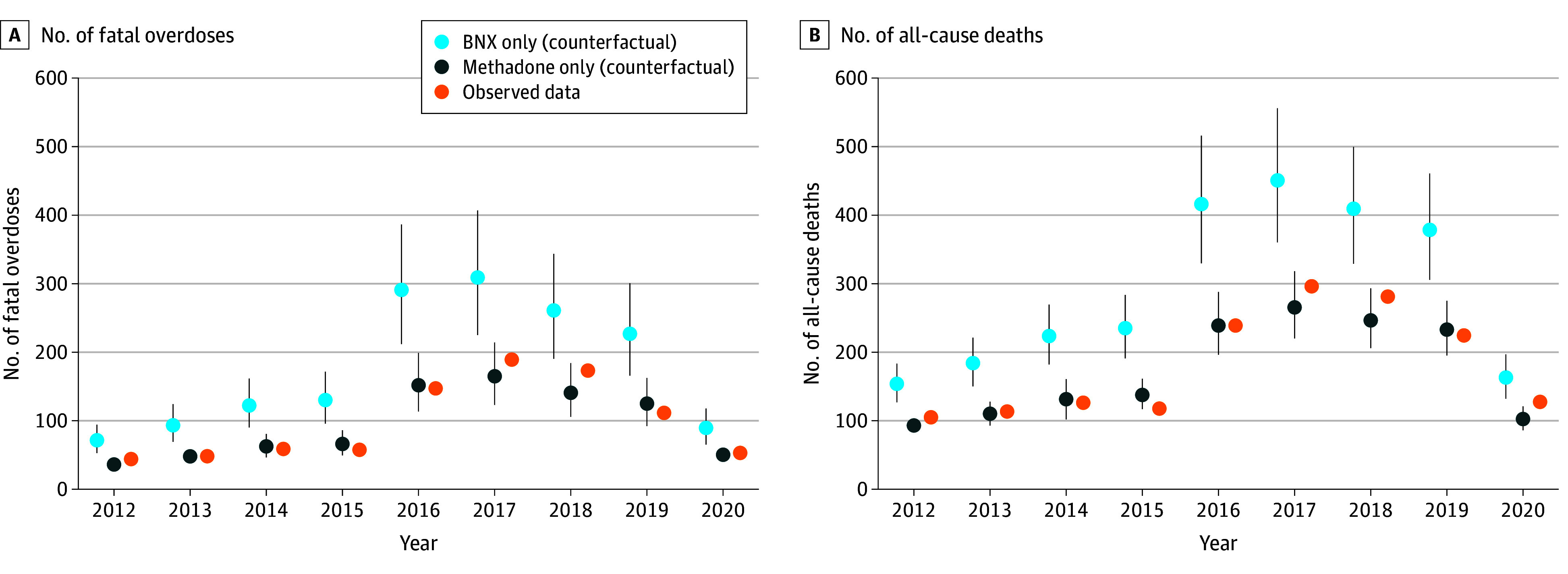
Model-Estimated Fatal Overdoses and All-Cause Deaths Compared With Observed Deaths Outcomes are shown for alternative treatment policies vs observed deaths and are from 2020 only up to March 17, 2020. Model outcomes are estimated from initiator analysis and derived from means and 95% credible intervals of 10 000 simulations. BNX indicates buprenorphine/naloxone; vertical lines, 95% credible intervals.

**Figure 2.**
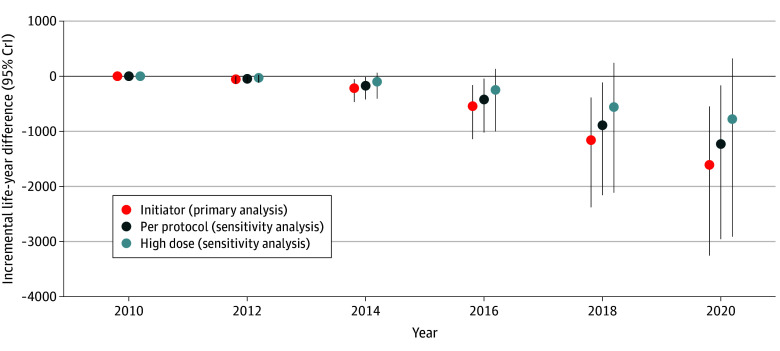
Cumulative Incremental Life-Years Associated With Alternative Treatment Policies Outcomes are shown for alternative buprenorphine/naloxone vs methadone treatment policies and are from 2020 only up to March 17, 2020. Results are cumulative over time and derived from means and 95% credible intervals (CrIs) of 10 000 simulations. Vertical lines indicate 95% CrIs.

Model-projected fatal overdoses and all-cause deaths for the population receiving exclusively methadone were closer to observed data (Figure 1) given that the status quo comprised a substantially higher proportion of individuals receiving methadone during most of the calibration period (eTable 5 in Supplement 1). We estimated that a policy of exclusively buprenorphine/naloxone would have −1602 incremental life-years (95% CrI, −3249 to −549 life-years; 1.6% decrease in total projected life-years) compared with methadone over our 10-year time horizon (Figure 2; eTable 6 and eFigure 9 in Supplement 1), with net health losses in nearly 100% of simulations (9998 simulations [99.98%]) (eTable 7 in Supplement 1). The population receiving exclusively buprenorphine/naloxone spent 4.18 of 9.52 life-years (43.9% of cumulative person-time) in treatment compared with 5.67 of 9.63 life-years (58.8%) for methadone (eFigures 10 and 11 in Supplement 1). Additionally, we estimated that individuals receiving exclusively buprenorphine/naloxone would experience an additional 221 fatal overdoses (95% CrI, 119 to 376 fatal overdoses) (eTable 8 in Supplement 1) and 303 all-cause deaths (95% CrI, 120 to 589 all-cause deaths) (eTable 9 in Supplement 1) compared with those receiving methadone.

### Sensitivity Analysis

In per-protocol analysis, we estimated that exclusively receiving buprenorphine/naloxone would have −1229 incremental life-years (95% CrI, −2959 to −168 incremental life-years) compared with receiving methadone (Figure 2; eTable 6 in Supplement 1), with net health losses in 9938 simulations (99.4%) (eTable 7 in Supplement 1). The population receiving exclusively buprenorphine/naloxone spent 3.51 of 9.51 life-years (36.9% of cumulative person-time) in treatment compared with 4.73 of 9.60 life-years (49.2%) for methadone (eFigures 12 and 13 in Supplement 1), with an additional 170 fatal overdoses (95% CrI, 42 to 382 fatal overdoses) (eTable 8 in Supplement 1) and 231 all-cause deaths (95% CrI, 39 to 542 all-cause deaths) (eTable 9 in Supplement 1) compared with the population receiving methadone.

Applying HRs from a subgroup of individuals receiving exclusively high daily dose buprenorphine/naloxone vs high daily dose methadone (571 of 10 107 clients with incident buprenorphine/naloxone [5.6%] and 430 of 15 507 clients with incident methadone [2.8%] received high daily doses), we estimated that exclusively buprenorphine/naloxone would have −776 incremental life-years (95% CrI, −2912 to 319 incremental life-years) vs methadone over 10 years (Figure 2; eTable 6 in Supplement 1), with net health losses in 8518 simulations (85.2%) (eTable 7 in Supplement 1). We estimated an additional 126 fatal overdoses (95% CrI, −9 to 391 fatal overdoses) (eTable 8 in Supplement 1) and 156 all-cause deaths (95% CrI, −46 to 549 all-cause deaths) (eTable 9 in Supplement 1) for the population receiving buprenorphine/naloxone compared with those receiving methadone.

**Figure 3.**
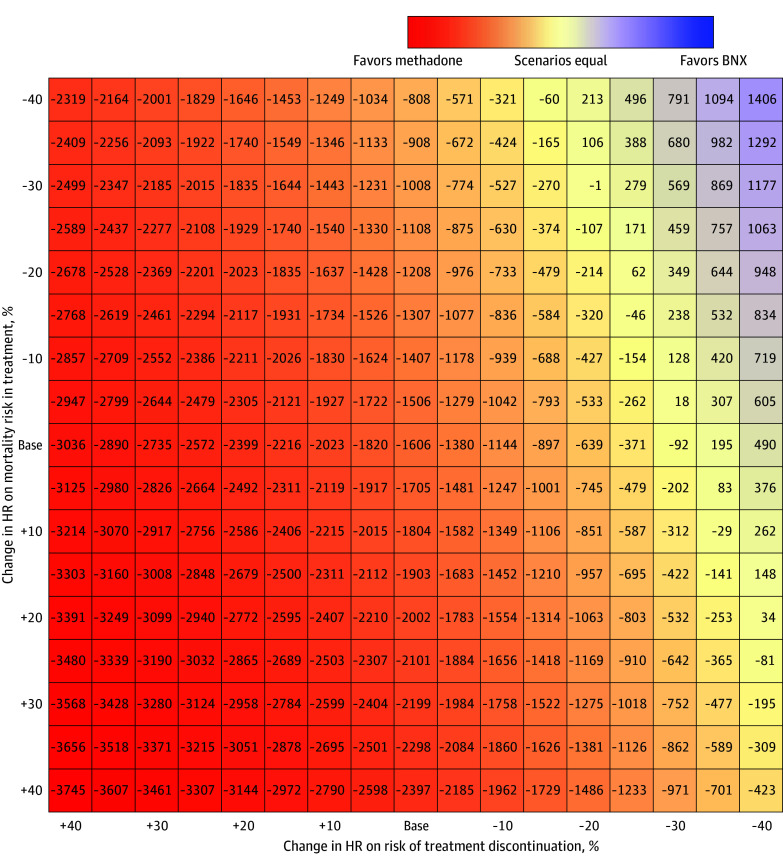
Two-Way Deterministic Sensitivity Analysis on Parameters for Risk of Treatment Discontinuation and Mortality in Treatment Model outcomes are cumulative incremental life-years from January 2010 to March 2020 for buprenorphine/naloxone (BNX) vs methadone alternative treatment policies from initiator analysis. HR indicates hazard ratio.

In 2-way sensitivity analysis on HRs for the risk of treatment discontinuation vs the risk of mortality while in treatment, a 40% reduction in the HR for risk of mortality (ie, improved mortality outcomes for buprenorphine/naloxone) still had net health losses for the population receiving exclusively buprenorphine/naloxone compared with those receiving methadone, holding treatment discontinuation constant (Figure 3). Holding risk of mortality constant, a 30% to 35% reduction in the hazard of treatment discontinuation for buprenorphine/naloxone vs methadone (ie, improved treatment retention for buprenorphine/naloxone) had net health gains.

Compared with our primary results, estimates for clients with incident OAT were more favorable to buprenorphine/naloxone; however, most simulations still had population health losses. Results for experienced clients with OAT were more favorable to methadone (eFigure 14 in Supplement 1). Among individual parameters varied in 1-way sensitivity analyses for our primary model specification (ie, at observed proportions of clients with incident OAT and experienced clients with OAT over time with all other parameters held constant), HRs for buprenorphine/naloxone vs methadone for the risk of mortality had the largest range of outcomes on incremental life-years when varied across their full uncertainty ranges (eFigure 15 in Supplement 1).

## Discussion

To our knowledge, this is the first decision analytical modeling study to estimate the comparative effectiveness of buprenorphine/naloxone vs methadone. This analysis followed from a population-based comparative effectiveness study of recipients of OAT in BC^21^ to estimate the combined association of differences in treatment retention and mortality with population-level health outcomes. We estimated that offering only buprenorphine/naloxone would have 1602 life-years lost (95% CrI, 549-3249 life-years lost) compared with methadone, with life-years lost in nearly all simulations. While estimates differed somewhat based on treatment definition and population subgroup, all results were directionally consistent.

Outcomes were influenced more by treatment retention than mortality risk while receiving treatment, and estimated differences in incremental life-years between treatments diverged more quickly after 2016 as fentanyl prevalence increased rapidly in the unregulated drug supply. HRs estimated from the prior statistical analysis for outcomes while receiving treatment were not meaningfully different after the introduction of fentanyl,^21^ but our model population was at increasingly greater risk of overdose and death when accounting for time spent out of treatment.

Results of subgroup and sensitivity analyses were overwhelmingly consistent with population-level results.^21^ Among individuals receiving high daily doses, outcomes were more favorable for those receiving buprenorphine/naloxone compared with methadone; however, we still estimated that this group would experience net health losses in 85.2% of simulations. Results from this subgroup were the most favorable to buprenorphine/naloxone among subgroups analyzed; however, they were generated from a small subset of the population (5.6% of clients with incident buprenorphine/naloxone and 2.8% of clients with incident methadone received high daily doses) and should be interpreted with caution. Given that other subgroups fell within these results and overall estimates, we did not include separate analyses for each subgroup.

While we estimated a wider range of outcomes from mortality HR parameters for buprenorphine/naloxone vs methadone in univariate sensitivity analysis, changes to HRs for the risk of treatment discontinuation were more influential on model outcomes when varied across the same relative ranges. This was a result of wider CIs for mortality parameters rather than overall impact. In general, parameter values associated with higher mortality risk while out of treatment favored methadone given that individuals spent more time in treatment.

### OAT Policy Implications

Our findings did not support recommendations of buprenorphine/naloxone as first-line treatment over methadone. In the most recent guideline update published in 2023, BC removed “first-line” terminology,^47^ although in the absence of patient preference or individual-level contraindicating factors, BC suggested that buprenorphine/naloxone may be favored vs other treatment options due to its safety profile. In the US, our findings support improving treatment access for methadone; this requires minimizing barriers, such as methadone prescription restrictions and insurance coverage, and expanding treatment availability to all health care settings.^48,49^ Similarly, regulations restricting the access of methadone solely to opioid treatment programs due to its classification as a Schedule II drug^10^ should be reassessed as more evidence for the benefits of relaxed prescribing and dispensing restrictions as a result of COVID-19 measures becomes available.^50,51,52^ Take-home dose extensions that were allowed during the COVID-19 pandemic as a temporary measure have now become permanent policy under part 8 of title 42 of the Code of Federal Regulations.^53^ In a time of unprecedented changes in the unregulated drug supply,^54^ access and choice of OUD treatment should ultimately be approached with a focus on shared decision-making.

### Limitations

Our study has several limitations related to data and assumptions. First, estimates of unregulated drug supply contamination used in the model were coarse and drawn from annual BC coroner data.^19^ These represented the best data available over the duration of our analytic period, and since the BC Centre on Substance Use began publishing drug-checking data in 2018, trends in fentanyl prevalence among suspected opioid samples have also been consistent with fentanyl prevalence among overdose deaths.^55^ Second, results from the per-protocol analysis should be interpreted with caution given the narrower definition of treatment, which meant that some individuals who were otherwise receiving OAT would be considered out of treatment due to suboptimal dosing or other censoring criteria. However, we felt it was important to include these results as a sensitivity analysis, particularly given that they were somewhat more favorable toward buprenorphine/naloxone. Additionally, given that comparative effectiveness HRs were drawn from a nonexperimental study, they may have been subject to uncontrolled confounding. Nevertheless, estimates were derived using both propensity score and instrumental variable approaches, which have different assumptions around unmeasured confounding, with consistent results. Third, we did not explicitly account for potential diversion of OAT medication in this analysis. Diversion of methadone has historically been cited as 1 of the reasons for more restrictive regulations compared with buprenorphine/naloxone,^52^ albeit with little empirical evidence to support this. However, for our period of analysis in BC, most methadone dispensations were provided via daily witnessed ingestion,^56^ so diversion was unlikely to be significant or overturn results of our analysis. Ultimately, our scope of analysis for this study was limited to a population of individuals with a diagnosed OUD who were accessing OAT through prescribers. Fourth, given that our analytic time horizon was limited to 10 years, our results likely understate differences between buprenorphine/naloxone vs methadone given that survival benefits would continue to accrue over the lifetime of our patient population.

## Conclusions

Results from this population-level decision analytical modeling study suggest that any advantages from an associated reduced risk of mortality while receiving treatment for buprenorphine/naloxone were outweighed by disadvantages in promoting treatment retention. Evaluated in a jurisdiction where both medications were available in office-based settings, our findings do not support recommendations of buprenorphine/naloxone as first-line treatment over methadone. While individual patients may have different likelihoods of benefitting from 1 treatment or another, individuals should ultimately be empowered to choose their treatment via shared decision-making with their clinician.

## Notes

Supplement 1. eAppendix 1. Model description eAppendix 2. Estimation of model parameters and data sources eAppendix 3. Model fitting and calibration eAppendix 4. Additional results eReferences. eTable 1. Population size and proportion of incident and experienced users of OAT in British Columbia, 2010-2020 eTable 2. Parameter distributions specified for probabilistic analysis draws eTable 3. Time-varying estimates of fentanyl prevalence, change in fentanyl prevalence, and probability of naloxone reversal eTable 4. Priors and posteriors for calibrated parameters eTable 5. Unique individuals initiating OAT episodes in each year eTable 6. Incremental life-years associated with alternative treatment policies of exclusively buprenorphine/naloxone vs exclusively methadone eTable 7. Percentage of simulations with incremental life years below threshold, 2010-2020 eTable 8. Incremental fatal overdoses from alternative treatment policies of exclusively buprenorphine/naloxone vs exclusively methadone eTable 9. Incremental all-cause deaths associated with alternative treatment policies of exclusively buprenorphine/naloxone vs exclusively methadone eFigure 1. Diagram of model health states and possible transitions eFigure 2. Comparison of fitted model status quo outputs with calibration targets (initiator analysis) eFigure 3. Comparison of fitted model status quo outputs with calibration targets (per-protocol analysis) eFigure 4. Prior and posterior distributions for calibrated model parameters (initiator analysis) eFigure 5. Posterior distributions and correlations for calibrated model parameters (initiator analysis) eFigure 6. Prior and posterior distributions for calibrated model parameters (per-protocol analysis) eFigure 7. Posterior distributions and correlations for calibrated model parameters (per-protocol analysis) eFigure 8. Plot of model-projected incident and experienced user population strata over time compared with observed data eFigure 9. Total cumulative life-years projected for each alternative treatment policy (primary analysis) eFigure 10. Model trace for buprenorphine/naloxone (initiator analysis) eFigure 11. Model trace for methadone (initiator analysis) eFigure 12. Model trace for buprenorphine/naloxone (per-protocol analysis) eFigure 13. Model trace for methadone (per-protocol analysis) eFigure 14. Cumulative incremental life-years for alternative buprenorphine/naloxone vs methadone treatment policies stratified by all incident and all experienced clients receiving OAT eFigure 15. Univariate sensitivity analysis on life-years gained associated with buprenorphine/naloxone vs methadone (initiator analysis)

Supplement 2.
Data Sharing Statement
