# Supplementary material for: Population-Level Health Benefits and Harms Associated With Buprenorphine/Naloxone vs Methadone
Source: JAMA Netw Open. 2025 Dec 26;8(12):e2551337. doi: 10.1001/jamanetworkopen.2025.51337 (PMC12743284; doi:10.1001/jamanetworkopen.2025.51337)
Supplement: Supplement 1. — eAppendix 1. Model description eAppendix 2. Estimation of model parameters and data sources eAppendix 3. Model fitting and calibration eAppendix 4. Additional results eReferences. eTable 1. Population size and proportion of incident and experienced users of OAT in British Columbia, 2010-2020 eTable 2. Parameter distributions specified for probabilistic analysis draws eTable 3. Time-varying estimates of fentanyl prevalence, change in fentanyl prevalence, and probability of naloxone reversal eTable 4. Priors and posteriors for calibrated parameters eTable 5. Unique individuals initiating OAT episodes in each year eTable 6. Incremental life-years associated with alternative treatment policies of exclusively buprenorphine/naloxone vs exclusively methadone eTable 7. Percentage of simulations with incremental life years below threshold, 2010-2020 eTable 8. Incremental fatal overdoses from alternative treatment policies of exclusively buprenorphine/naloxone vs exclusively methadone eTable 9. Incremental all-cause deaths associated with alternative treatment policies of exclusively buprenorphine/naloxone vs exclusively methadone eFigure 1. Diagram of model health states and possible transitions eFigure 2. Comparison of fitted model status quo outputs with calibration targets (initiator analysis) eFigure 3. Comparison of fitted model status quo outputs with calibration targets (per-protocol analysis) eFigure 4. Prior and posterior distributions for calibrated model parameters (initiator analysis) eFigure 5. Posterior distributions and correlations for calibrated model parameters (initiator analysis) eFigure 6. Prior and posterior distributions for calibrated model parameters (per-protocol analysis) eFigure 7. Posterior distributions and correlations for calibrated model parameters (per-protocol analysis) eFigure 8. Plot of model-projected incident and experienced user population strata over time compared with observed data eFigure 9. Total cumulative life-years projected for [file jamanetwopen-e2551337-s001.pdf]

Supplemental Online Content

Enns B, Guerra-Alejos BC, Min JE, Carter A, Siebert U, Nosyk B. Population-level health benefits and harms associated with buprenorphine/naloxone vs methadone. *JAMA Netw Open*. 2025;8(12):e2551337. doi:10.1001/jamanetworkopen.2025.51337

eAppendix 1. Model description

eAppendix 2. Estimation of model parameters and data sources

eAppendix 3. Model fitting and calibration

eAppendix 4. Additional results

eReferences.

eTable 1. Population size and proportion of incident and experienced users of OAT in British Columbia, 2010-2020

eTable 2. Parameter distributions specified for probabilistic analysis draws

eTable 3. Time-varying estimates of fentanyl prevalence, change in fentanyl prevalence, and probability of naloxone reversal

eTable 4. Priors and posteriors for calibrated parameters

eTable 5. Unique individuals initiating OAT episodes in each year

eTable 6. Incremental life-years associated with alternative treatment policies of exclusively buprenorphine/naloxone vs exclusively methadone

eTable 7. Percentage of simulations with incremental life years below threshold, 2010-2020

eTable 8. Incremental fatal overdoses from alternative treatment policies of exclusively buprenorphine/naloxone vs exclusively methadone

eTable 9. Incremental all-cause deaths associated with alternative treatment policies of exclusively buprenorphine/naloxone vs exclusively methadone

eFigure 1. Diagram of model health states and possible transitions

eFigure 2. Comparison of fitted model status quo outputs with calibration targets (initiator analysis)

eFigure 3. Comparison of fitted model status quo outputs with calibration targets (per-protocol analysis)

eFigure 4. Prior and posterior distributions for calibrated model parameters (initiator analysis)

eFigure 5. Posterior distributions and correlations for calibrated model parameters (initiator analysis)

eFigure 6. Prior and posterior distributions for calibrated model parameters (per-protocol analysis)

eFigure 7. Posterior distributions and correlations for calibrated model parameters (per-protocol analysis)

eFigure 8. Plot of model-projected incident and experienced user population strata over time compared with observed data

© 2025 Enns B et al. *JAMA Network Open*.

eFigure 9. Total cumulative life-years projected for each alternative treatment policy (primary analysis)

eFigure 10. Model trace for buprenorphine/naloxone (initiator analysis)

eFigure 11. Model trace for methadone (initiator analysis)

eFigure 12. Model trace for buprenorphine/naloxone (per-protocol analysis)

eFigure 13. Model trace for methadone (per-protocol analysis)

eFigure 14. Cumulative incremental life-years for alternative buprenorphine/naloxone vs methadone treatment policies stratified by all incident and all experienced clients receiving OAT

eFigure 15. Univariate sensitivity analysis on life-years gained associated with buprenorphine/naloxone vs methadone (initiator analysis)

This supplemental material has been provided by the authors to give readers additional information about their work.

© 2025 Enns B et al. *JAMA Network Open*.

eAppendix 1. Model description

For this analysis, we adapted a semi-Markov cohort model, which incorporated both patient history and calendar-time. This model was previously used to estimate the cost-effectiveness of diacetylmorphine versus methadone in British Columbia,[1] the cost-effectiveness of publicly-funded opioid agonist treatment (OAT) in California,[2] and the cost-effectiveness of flexible take-home buprenorphine/naloxone versus methadone in Canada, alongside the OPTIMA trial.[3] This analysis followed from a recent large-scale comparative effectiveness study,[4] to estimate the population-level comparative effectiveness of buprenorphine/naloxone versus methadone for British Columbia (BC), Canada. We estimated incremental life years between hypothetical populations of individuals accessing exclusively buprenorphine/naloxone, or only methadone. The model included six mutually exclusive health states: buprenorphine/naloxone, methadone, out-of-treatment, long-term abstinence, overdose, and death, stratified by individuals in their first OAT episode ('incident OAT clients') and those in their second episode or higher ('experienced OAT clients'), to incorporate estimated differences in outcomes between these two groups.[4] Individuals entered the model in either buprenorphine/naloxone or methadone, and could move between health states, or die, in weekly cycles. As this analysis was not conducted on a fixed cohort followed over a lifetime, model outcomes were scaled annually by the number of individuals observed in our data in a given year, to reflect the increasing number of individuals presenting for treatment over time.

Time-dependent model parameters

We incorporated two types of time dependence in the model: (i) state-residence; and (ii) calendar-time. For state-residence time dependence, the probability of remaining in (or discontinuing) health states was based on empirical estimates on time to discontinuation (Weibull regression estimates to calculate the baseline hazard function and time-dependent probability of discontinuing from a given health state, conditional on time spent in that state), using a three-dimensional array to account for the number of cycles spent in a given health state. In order to program time-dependence within health states, we employed methods described in Hawkins et al,[5] who provide a method of breaking up arrays, doing calculations piecemeal, and achieving the correct weighted averages. For calendar-time dependence, we allowed for parameters calculating the risk of overdose and mortality to be updated annually, incorporating observed changes in fentanyl prevalence, and naloxone availability.

Calculating the probability of overdose

Transition probabilities to overdose (risk of overdose) were calculated from base overdose rates for each health state, with the probability of fatal overdose determined via fatal overdose rate conditional on overdose, multiplied by the estimated probability of naloxone reversal. Individuals faced a probability of overdose each week, which varied depending on their current health state, risk of fentanyl exposure (annual estimated prevalence and year-over-year change in prevalence), and if it was their first week following treatment discontinuation. If individuals experienced a non-fatal overdose, they could continue in the model and return to treatment, remain out of treatment, or overdose again in the following week.

© 2025 Enns B et al. *JAMA Network Open*.

We incorporated state-specific overdose probabilities in the model as rates per person-week with multipliers for elevated overdose risk in the first week of a given health state, and multipliers that were a function of the estimated fentanyl prevalence and annual change in prevalence in the unregulated opioid supply. Weekly rates were converted into weekly probabilities using the following equation.

$$(1)\ P_{overdose} = 1 - e^{(-base\ rate\ *\ conditional\ multipliers)}$$

The probability of overdose accounted for the probability of fentanyl exposure and elevated risk of overdose when exposed to fentanyl, along with the base overdose probability in the absence of fentanyl which we calibrated in the model. We distinguished the first week of opioid use following treatment discontinuation with a higher transition probability to overdose than subsequent weeks, based on evidence showing that individuals have an elevated overdose risk in period immediately following treatment discontinuation.[6–8] We included this parameter as a multiplier on the rate of overdose in the first week out of treatment.[9]

Impact of fentanyl on overdose

The probability of overdose was influenced by both the overall potency (percentage of unregulated drug poisoning deaths in British Columbia with fentanyl detected), and unpredictability (year-over-year change in fentanyl prevalence) of the unregulated drug supply. We incorporated the effect of fentanyl on overdose via an exponential function, including separate multipliers on both the overall prevalence (equation 2) and change in fentanyl prevalence (equation 3), which were calibrated in the model.

$$(2)\ Multiplier\ (fentanyl\ prevalence)_{year} = e^{(prevalence\ multiplier\ *\ fentanyl\ prevalence_{year})}$$

$$(3)\ Multiplier\ (fentanyl\ \Delta)_{year} = e^{(\Delta\ multiplier\ *\ fentanyl\ \Delta_{year})}$$

Probability of fatal overdose

We estimated a base rate of fatal overdose (conditional on overdose) via model calibration, with multipliers for the increased risk of fatal overdose when individuals were out-of-treatment versus in-treatment, adjusted for the estimated annual probability of naloxone reversal.

$$(4)\ P_{fatal\ overdose} = [1 - e^{(-fatal\ overdose\ rate\ *\ multipliers)}] * (1 - P_{NX\ rev})$$

Comparative effectiveness of buprenorphine/naloxone versus methadone

Differences between model populations in alternative treatment policies were parameterized in the model using hazard ratio estimates,[4] on the risk of treatment discontinuation, and risk of mortality, for buprenorphine/naloxone compared to methadone. To match the mutually exclusive strata defined in our model, we used modified estimates for OAT-experienced users, which excluded those in their first treatment episode. We used per-protocol estimates for mortality hazard ratios in both model specifications, as the initiator definition was not compatible with estimating mortality differences for individuals engaged in treatment. Comparative effectiveness hazard ratio estimates on treatment discontinuation for buprenorphine/naloxone versus methadone were used

© 2025 Enns B et al. *JAMA Network Open*.

to modify the base hazard rates for treatment discontinuation estimated for methadone, which were then used to calculate time-dependent weekly probabilities of treatment discontinuation for individuals on buprenorphine/naloxone. We used the following equation, as detailed in Chapter 3 of Briggs et al:[10]

$$(5)\ P_{remain} = e^{(HR * \lambda * ((t-1)^{\gamma} - t^{\gamma})}$$

Where HR is a modifier to the base hazard rate estimate for a given intervention (in this case, HR refers to the comparative effectiveness hazard ratio on discontinuation for buprenorphine/naloxone relative to methadone, with methadone as the base estimate, where an HR = 1 would be equivalent), λ is the scale parameter, and γ the shape parameter of the Weibull distribution. We required the probability of remaining as a model input, rather than discontinuation, so the equation was modified to give: 1 – probability of discontinuation.

Comparative effectiveness estimates on risk of mortality for buprenorphine/naloxone were applied directly to mortality rates for individuals in treatment (fatal overdose and non-overdose mortality rates while in treatment, including mortality in the first week post-discontinuation), which were then used to derive mortality probabilities. Apart from differences in treatment retention and mortality in treatment established via comparative effectiveness estimates, all other model parameters for populations in each alternative treatment policy were identical.

© 2025 Enns B et al. *JAMA Network Open*.

eAppendix 2. Estimation of model parameters and data sources

Our primary data source was population-level longitudinal health administrative data from all individuals with OUD in British Columbia. General information on the population-level cohort has been published previously,[11] along details on data set construction specific to this analysis.[4] Briefly, our data capture included all OAT recipients between January 1, 2010 and March 17, 2020 from nine linked health-administrative databases: Medical Services Plan (MSP),[12] Discharge Abstract Database (DAD),[13] PharmaNet,[14] Vital Statistics,[15] BC Corrections,[16] National Ambulatory Care Reporting System (NACRS),[17] BC Perinatal database,[18] Client roster,[19] and Social Development and Poverty Reduction (SDPR).[20] Data after March 17, 2020 were excluded due to the provincial COVID-19 pandemic emergency declaration. Our sample for this analysis included 40,461 individuals.

Other data sources

We derived model inputs for fentanyl prevalence from BC Coroner's service data,[21] using the annual percentage of unregulated drug poisoning deaths with fentanyl detected. We incorporated data on ambulance-attended overdoses from BC Emergency Health Services,[22] to help estimate the annual number of non-fatal overdoses. We derived age-specific general population mortality estimates from Statistics Canada for British Columbia.[23] We used data from Irvine et al,[24] and Lei et al[25] to estimate parameters for the probability of naloxone reversal following an overdose. We used estimates from Durand et al[9] for multipliers of overdose rates in the first week following treatment discontinuation. We also supplemented information on ambulance-attended overdoses with literature sources, to adjust for the estimated proportion of overdoses that were captured in EHS data.[26]

Model parameterization and data

We conducted primary data analyses, and calibrated the model, using two different treatment definitions in our data set: 'initiator' analysis, estimated via propensity score matching, and 'per-protocol' analysis.[4] In initiator analysis, individuals were followed from OAT initiation to discontinuation, death, or end of study follow-up (24 months or Mar 17, 2020), whichever occurred earlier. Treatment was based on medications assigned at initiation regardless of treatment adherence. In per-protocol analysis, treatment was defined according to dosing guidelines, with episodes discontinued when dosing became suboptimal or another censoring event occurred such as medication discontinuation, medication switch or ineligibility for study entry (pregnancy, taper initiation, incarceration or receipt of cancer or palliative care).

Health state definitions

Individuals were considered in treatment based on initiator or per-protocol specifications described above, and otherwise were classified as out-of-treatment. We defined 'long-term abstinence' for those individuals who were out of treatment, but with evidence of a completed taper off of OAT medication and at least 18-months of no OUD-related health records.

© 2025 Enns B et al. *JAMA Network Open*.

Probability of remaining in health state

When constructing the state-residence time-dependent transition array, probabilities of overdose, fatal overdose and non-overdose death were populated first. Then, conditional on remaining alive and not overdosing, time-dependent probabilities of remaining in their current health state were calculated, using Weibull regression estimates or calibrated parameters. We estimated Weibull parameters from our primary analytic data set based on estimated time to discontinuation, and calibrated state-residence time-dependent parameters for the probability of remaining out-of-treatment and in long-term abstinence, to match targets for the observed percentage of time spent out of treatment in each year. Priors were estimated from our data, and calibrated to ensure that model estimates matched observed surveillance data. In the comparative effectiveness analysis, we estimated Weibull parameters for the probability of remaining in methadone, and applied hazard ratio estimates to derive the risk of discontinuation for buprenorphine/naloxone. All treatment retention parameters were estimated relative to risk of discontinuation, with the probability of remaining calculated as: 1 – probability of discontinuation.

Health state transitions after discontinuation

Conditional on discontinuation, we estimated transitions to other, non-overdose, health states using the observed state transition proportions after health state discontinuation in our population-level data. We assumed that individuals would only reach long-term opioid abstinence through treatment, and individuals exiting long-term abstinence would not go directly into treatment (i.e., would first transition to overdose or out-of-treatment).

Overdose

Base weekly rates of overdose, fatal overdose, and non-overdose death were estimated from our population-level data, using estimates for 2010 – 2013 for priors, when fentanyl prevalence was still below 15% in overdose deaths. We calibrated these rates in the model, incorporating annual data on fentanyl prevalence and naloxone distribution. We estimated the risk of overdose and mortality for out-of-treatment versus OAT via multiplicative rate ratios on rates in OAT, estimated from our data, using the average rate ratios for fatal overdose and drug-related acute care visits from 2010 – 2020. Out-of-treatment versus OAT rate ratios for overdose and fatal overdose were given log-normal distributions in our probabilistic analysis, using the mean and outer range of estimates between 2010 and 2020 to parameterize the distribution. We used literature estimates to derive rate ratios for the increase in overdose risk in the first week following treatment discontinuation, relative to week 2+.[9] We incorporated annual fentanyl prevalence data from the BC Coroner's Service (percentage of unregulated drug poisoning deaths with fentanyl detected).[27] We calculated the probability of naloxone reversal using estimates from 2017 in British Columbia as baseline,[24] and 2012 – 2016 using estimated overdose reversals reported by the BC CDC [28] relative to 2017, and estimates for 2018 – 2020 for the adjusted odds of using a kit relative to 2017 (**eTable 3**).[25]

Non-overdose mortality

© 2025 Enns B et al. *JAMA Network Open*.

Base estimates for risk of non-overdose mortality were drawn from population-level estimates for the province of British Columbia.[23] Age- and sex-adjusted annual mortality rates were obtained from statistical life tables, and converted to monthly non-overdose death transition probabilities from each state using the formula:

$$(6)\ P_{non-od\ mortality} = 1 - e^{\left(-annual\ mortality\ rate * \left(\frac{1}{52}\right) * multiplier\right)}$$

We held the age-adjusted background mortality risk constant at 33 years throughout the analytic period, to match the median age of our study population over that time period. We assumed that mortality risk for individuals in long-term opioid abstinence was equivalent to that of the general population at the same age and gender mix. We estimated rate ratio multipliers for non-overdose mortality (OAT relative to general population and out-of-treatment relative to OAT) from our data. We used the average and standard error of annual rate ratios for non-overdose mortality (2010 – 2020) for the out-of-treatment versus OAT non-overdose mortality parameter. We calibrated non-overdose mortality rate ratios for OAT versus general population, with priors estimated from non-overdose mortality rates observed among people on OAT versus overall population mortality. We calibrated to observed numbers of non-overdose deaths in our population, and compared model outputs to observed data. Rate ratios for non-overdose mortality were given log-normal distributions in our probabilistic analysis, using the mean and outer range of yearly estimates between 2010 and 2020 to parameterize the distribution.

© 2025 Enns B et al. *JAMA Network Open*.

eAppendix 3. Model fitting and calibration

We used a Bayesian approach to calibrate overdose, mortality, and parameters to calculate the probability of remaining out of treatment to five different targets, incorporating annual data on estimated fentanyl prevalence in the illicit drug supply and probability of overdose reversal via naloxone. We constructed a likelihood function to match model outputs to target data estimates from 2012 through 2020, to capture the first year of fentanyl detection in illicit drug poisoning deaths in 2012. Prior to conducting our counterfactual alternative treatment policy analysis, we needed to establish the underlying overdose risk over time, accounting for changes in the unregulated drug supply, as well as the scale up of take-home naloxone in BC. We did not distinguish treatment types in calibration, but rather the underlying risks for individuals in OAT treatment versus out-of-treatment. We calibrated model parameters for initiator and per-protocol analysis separately, to the same target data, as treatment status was defined differently (initiator analysis included some individuals in treatment who would be censored for non-guideline-concordant treatment under the per-protocol definition and classified as out-of-treatment).

Parameter selection for calibration

We chose to calibrate parameters for estimating the background probability of overdose, overdose death, and non-overdose death; including (i) weekly overdose rate in OAT, (ii) fatal overdose rate in OAT, increased risk of overdose from (iii) overall fentanyl prevalence, and (iv) year-over-year change in fentanyl prevalence, (v) increased risk of non-overdose mortality while in OAT (relative to the general population), (vi) Weibull distribution shape and scale parameters for determining the time-varying probability of remaining out of treatment.

Specification of priors

We set priors for overdose and fatal overdose rates based on population-level rates of drug-related acute care visits (per-person-week, using average rate estimates from 2010-2013 which pre-dated the increase in fentanyl, take-home naloxone scale-up, and public health emergency), estimated from population-level BC OAT data (**eTable 4**). Similarly, priors on fatal overdose rates were derived from relative rates of fatal overdose to drug-related acute care visits for individuals in and out of OAT treatment. Priors on hazard ratios for non-overdose death were estimated from mortality rates observed in our data compared to general population mortality. For calculating the probability of remaining out of treatment or in long-term abstinence, we specified Weibull shape priors as 1 (assuming constant over time), and scale priors based on regression estimates from our data. Finally, we specified weakly informative priors on overdose rate multipliers for fentanyl prevalence and change in fentanyl prevalence, assuming that both would be positively related to overdose risk.

Calibration targets

We calibrated the model to annual data on: (i) fatal overdoses, (ii) non-overdose deaths; (iii) proportion of time spent out of treatment; (iv) proportion of time spent in long-term abstinence; and (v) estimated number of non-fatal overdoses (approximated using ambulance-attended overdoses and inflated for underreporting using data

© 2025 Enns B et al. *JAMA Network Open*.

from literature sources), for 2012 through 2020. We chose this time frame to capture the first detection of fentanyl in overdose deaths in 2012, as well as the increase in take-home naloxone availability beginning in 2016 (**eTable 3**). We estimated fatal overdoses and non-overdose deaths using the rate per person-years of observation in our data, converted into an annual proportion. We estimated the relative proportion of time spent in and out of treatment based on person-time spent in and out of treatment in each year, as well as the proportion of time observed in long-term abstinence. Given the uncertainty in estimating non-fatal overdoses from drug-related acute care visits and ambulance-attended overdoses – and likelihood of underreporting – we weighted this target at 50% of other targets when defining the objective function.

Calibration procedure

We calibrated the model using a Bayesian approach. Modern Bayesian calibration algorithms improve efficiency by concentrating sampling of parameter values in regions of the parameter space with higher posterior density. We used an incremental mixture importance sampling (IMIS) algorithm to calibrate the model,[29,30] which has been shown to be more computationally efficient (producing a larger effective sample size (ESS) relative to total number of function evaluations) than other algorithms.[31]

© 2025 Enns B et al. *JAMA Network Open*.

eAppendix 4. Additional results

Model calibration

In model calibration we obtained a posterior sample size of 10,000. The posterior sample generated an ESS of 3694 and 3794, with 6402 and 6314 unique parameter sets for initiator and per-protocol analyses, respectively. We ran the model for each joint posterior sample, and evaluated its fit to our targets using the mean and 95% credible interval of 10,000 samples. Model outputs fit well to all targets, particularly fatal overdoses and non-overdose deaths (**eFigures 2 and 3**). The inclusion of overdose risk multipliers for both fentanyl prevalence and change allowed the model to capture the rapid increase in overdose deaths beginning in 2015. The annual number of fatal overdoses were a result of many idiosyncratic factors that are not fully captured in surveillance data. While model-estimated fatal overdoses were somewhat lower in peak years of 2017 and 2018 compared to observed data targets, outputs still fit well across all years of calibration. Model-estimated non-fatal overdoses captured the increasing trend, but at higher overall levels than our targets, beginning in 2016. Calibration target estimates for non-fatal overdoses were highly uncertain, and likely to be underestimated compared to the actual number of non-fatal overdoses. The rapid scale-up of naloxone availability beginning in 2016, as well as other harm reduction interventions, meant that more overdoses could be reversed and managed without necessarily interacting with the health care system, and thus may not be reflected in EMS or acute care surveillance data. Plots of posterior calibrated parameter distributions versus priors, and correlations for joint parameter distributions are shown in **eFigures 4 and 5** (initiator analysis), and **eFigures 6 and 7** (per-protocol analysis). Overdose parameter posteriors were generally calibrated to slightly lower values than their prior means (**eTable 4**), although parameter estimates were not substantially different after calibration. Narrower posterior distributions for all parameters relative to their prior distributions indicated that calibration target data was informative in identifying all parameters.

Model trace

Model trace plots from deterministic analyses of buprenorphine/naloxone and methadone alternative treatment policies are shown in **eFigures 10 and 11** (initiator analysis), and **eFigures 12 and 13** (per-protocol analysis). In both specifications, the population receiving exclusively methadone spent more time in treatment compared to the exclusively buprenorphine/naloxone, 58.8% vs. 43.9% in initiator analysis, and 49.2% vs. 36.9% in per-protocol analysis. As a result of a narrower definition of treatment, overall time spent in treatment was lower in per-protocol analysis compared to initiator analysis, and the proportion of time spent in treatment was declining over time, which was consistent with overall trends. The plots also highlight the increase in overdoses and fatal overdoses beginning in 2016. It should be noted that fatal overdoses (and non-overdose deaths) in the model trace plots were a cumulative total over time, while non-fatal overdoses were specific to each weekly time point. Different out-of-treatment ('OOT') health states ('OOT', 'OOT-B', and 'OOT-M') shown in trace plots were introduced for the purposes of tracking individuals in the model, and were functionally equivalent after the first week of state-residence time. These were added so that we could capture the difference in first-week mortality

© 2025 Enns B et al. *JAMA Network Open*.

risk between OOT-B (out-of-treatment following discontinuation from buprenorphine/naloxone) and OOT-M (out-of-treatment following discontinuation from methadone), where comparative effectiveness hazard ratios for risk of mortality in treatment were applied to first week overdose mortality following discontinuation from buprenorphine/naloxone (OOT-B).

© 2025 Enns B et al. *JAMA Network Open*.

eReferences

1. Nosyk B, Guh DP, Bansback NJ, et al. Cost-effectiveness of diacetylmorphine versus methadone for chronic opioid dependence refractory to treatment. *CMAJ*. 2012;184(6):E317-28. doi:10.1503/cmaj.110669

2. Krebs E, Enns B, Evans E, et al. Cost-Effectiveness of Publicly Funded Treatment of Opioid Use Disorder in California. *Ann Intern Med*. 2018;168(1):10-19. doi:10.7326/M17-0611

3. Enns B, Krebs E, Whitehurst DGT, et al. Cost-effectiveness of flexible take-home buprenorphine-naloxone versus methadone for treatment of prescription-type opioid use disorder. *Drug Alcohol Depend*. 2023;247:109893. doi:10.1016/j.drugalcdep.2023.109893

4. Nosyk B SM Min JE, Homayra F, Kurz M, Guerra Alejos BC, Yan R, Piske M, Seaman SR, Bach P, Greenland S, Karim ME, Siebert U, Bruneau J, Gustafson P, Kampman K, Korthuis PT, Loughin T, McCandless LC, Platt RW, Schnepel KT. Buprenorphine/Naloxone vs Methadone for the Treatment of Opioid Use Disorder. *JAMA*. Published online 2024. doi:10.1001/jama.2024.16954

5. Hawkins N, Sculpher M, Epstein D. Cost-effectiveness analysis of treatments for chronic disease: using R to incorporate time dependency of treatment response. *Med Decis Mak Int J Soc Med Decis Mak*. 2005;25(5):511-519. doi:10.1177/0272989X05280562

6. Pearce LA, Min JE, Piske M, al et. Opioid agonist treatment and risk of mortality during opioid overdose public health emergency: population based retrospective cohort study. *BMJ*. 2020;368:m772. doi:10.1136/bmj.m772

7. Evans E, Li L, Min J, et al. Mortality among individuals accessing pharmacological treatment for opioid dependence in California, 2006-10. *Addict Abingdon Engl*. 2015;110(6):996-1005. doi:10.1111/add.12863

8. Sordo L, Barrio G, Bravo MJ, et al. Mortality risk during and after opioid substitution treatment: systematic review and meta-analysis of cohort studies. *BMJ*. 2017;357:j1550. doi:10.1136/bmj.j1550

9. Durand L, Keenan E, Boland F, et al. Consensus recommendations for opioid agonist treatment following the introduction of emergency clinical guidelines in Ireland during the COVID-19 pandemic: A national Delphi study. *Int J Drug Policy*. 2022;106:103768. doi:10.1016/j.drugpo.2022.103768

10. Caldwell D. Decision Modelling for Health Economic Evaluation. A Briggs, M Sculpher, K Claxton. *Int J Epidemiol*. 2007;36(2):476-477. doi:10.1093/ije/dym062

11. Homayra F, Pearce LA, Wang L, et al. Cohort profile: The provincial substance use disorder cohort in British Columbia, Canada. *Int J Epidemiol*. 2021;49(6):1776. doi:10.1093/ije/dyaa150

12. British Columbia Ministry of Health. Data from: Medical Services Plan (MSP) Payment Information File. Published online 2022. http://www.health.gov.bc.ca/data/

13. Canadian Institute for Health Information. Data from: Discharge Abstract Database (Hospital Separations). Published online 2022. http://www.health.gov.bc.ca/data/

14. British Columbia Ministry of Health. Data from: PharmaNet. Published online 2022. http://www.health.gov.bc.ca/data/

15. British Columbia Ministry of Health. Data from: Vital Statistics Deaths. Published online 2022. http://www.health.gov.bc.ca/data/

16. Ministry of Public Safety and Solicitor General (PSSG). Data from: BC Corrections Dataset. Published online 2022. http://www.health.gov.bc.ca/data/

© 2025 Enns B et al. *JAMA Network Open*.

17. British Columbia Ministry of Health. Data from: National Ambulatory Care Reporting System (NACRS). Published online 2022. http://www.health.gov.bc.ca/data/

18. Perinatal Services BC. Data from: British Columbia Perinatal Data Registry. Published online 2022. http://www.health.gov.bc.ca/data/

19. British Columbia Ministry of Health. Data from: Client Roster. Published online 2022. http://www.health.gov.bc.ca/data/

20. British Columbia Ministry of Social Development and Poverty Reduction. Data from: Social Development and Poverty Reduction Database (SDPR). Published online 2022. http://www.health.gov.bc.ca/data/

21. British Columbia Coroners Service. Unregulated Drug Deaths - Drugs Involved. Published online 2024. https://app.powerbi.com/view?r=eyJrIjoiMmIzN2UyYTItNmM0Yi00MGZhLWI1NDUtOTRhZGNjYTBhZDJlIiwidCI6IjZmZGI1MjAwLTNkMGQtNGE4YS1iMDM2LWQzNjg1ZTM1OWFkYyJ9

22. BC Emergency Health Services. Overdose and Drug Poisoning Data. Published online 2025. https://www.bcehs.ca/about/accountability/data/overdose-drug-poisoning-data

23. Statistics Canada. *Life Tables, Canada, Provinces and Territories 1980/1982 to 2020/2022 (Three-Year Estimates), and 1980 to 2022 (Single-Year Estimates)*. Statistics Canada; 2023. https://www150.statcan.gc.ca/n1/pub/84-537-x/84-537-x2023002-eng.pdf

24. Irvine MA, Kuo M, Buxton JA, et al. Modelling the combined impact of interventions in averting deaths during a synthetic-opioid overdose epidemic. *Addict Abingdon Engl*. 2019;114(9):1602-1613. doi:10.1111/add.14664

25. Lei V, Ferguson M, Geiger R, Williams S, Liu L, Buxton JA. Factors associated with take-home naloxone kit usage in British Columbia: an analysis of administrative data. *Subst Abuse Treat Prev Policy*. 2022;17(1):25. doi:10.1186/s13011-022-00452-8

26. MacDougall L, Smolina K, Otterstatter M, et al. Development and characteristics of the Provincial Overdose Cohort in British Columbia, Canada. *PloS One*. 2019;14(1):e0210129. doi:10.1371/journal.pone.0210129

27. British Columbia Coroner’s Service. *Illicit Drug Toxicity Deaths in BC, January 1, 2012 - December 31, 2022*. British Columbia Coroner’s Service; 2023. https://www2.gov.bc.ca/assets/gov/birth-adoption-death-marriage-and-divorce/deaths/coroners-service/statistical/illicit-drug.pdf

28. Toward the Heart: BC CDC Harm Reduction Services. Take Home Naloxone Program. Published online January 31, 2025. https://towardtheheart.com/naloxone

29. Steele RJ, Raftery AE, Emond MJ. Computing Normalizing Constants for Finite Mixture Models via Incremental Mixture Importance Sampling (IMIS). *J Comput Graph Stat*. 2006;15(3):712-734. doi:10.1198/106186006X132358

30. Raftery AE, Bao L. Estimating and Projecting Trends in HIV/AIDS Generalized Epidemics Using Incremental Mixture Importance Sampling. *Biometrics*. 2010;66(4):1162-1173. doi:10.1111/j.1541-0420.2010.01399.x

31. Menzies NA, Soeteman DI, Pandya A, Kim JJ. Bayesian Methods for Calibrating Health Policy Models: A Tutorial. *PharmacoEconomics*. 2017;35(6):613-624. doi:10.1007/s40273-017-0494-4

© 2025 Enns B et al. *JAMA Network Open*.

eTable 1. Population size and proportion of incident and experienced users of OAT in British Columbia, from 2010 – 2020

Year	Number of individuals (population scaling)	Proportion incident OAT clients (%)	Proportion experienced OAT clients (%)
2010	4559	48.2%	51.8%
2011	6999	47.6%	52.4%
2012	8757	46.6%	53.4%
2013	9104	42.4%	57.6%
2014	10186	39.3%	60.7%
2015	10569	38.3%	61.7%
2016	12600	40.3%	59.7%
2017	14365	39.3%	60.7%
2018	14923	36.8%	63.2%
2019	15216	33.0%	67.0%
2020[a]	7390	28.7%	71.3%

a. Outcomes from 2020 only up to March 17, 2020. OAT: opioid agonist treatment.

© 2025 Enns B et al. *JAMA Network Open*.

eTable 2. Parameter distributions specified for probabilistic analysis draws

Parameter type	Data Requirement	Distribution	Description
Remain in given health state	Point estimate, CI	Weibull	To calculate weekly probability of remaining in each health state
Comparative effectiveness parameters	Point estimate, CI	Lognormal	Hazard ratios on risk of treatment discontinuation and mortality in treatment for buprenorphine/naloxone vs. methadone
Transition to (from) health states	Number of individuals in each category	Dirichlet/Beta	Proportional distribution of individuals transitioning out of given health state into possible destination states
Calibrated parameters	Posterior distribution	Joint posterior	Values sampled from empirically-derived joint posterior distributions post-calibration
Non-calibrated overdose and mortality parameters	Point estimate, CI	Lognormal	Overdose, fatal overdose, and non-overdose mortality rate multipliers

© 2025 Enns B et al. *JAMA Network Open*.

eTable 3. Time-varying estimates of fentanyl prevalence, change in fentanyl prevalence and probability of naloxone reversal

Year	Fentanyl prevalence	Absolute change in fentanyl prevalence	Probability of naloxone reversal
2010	0.0%	0.0%	0.0%
2011	0.0%	0.0%	0.0%
2012	4.0%	4.0%	0.0%
2013	15.0%	11.0%	0.2%
2014	25.0%	10.0%	0.8%
2015	29.0%	4.0%	2.6%
2016	67.0%	38.0%	25.4%
2017	82.0%	15.0%	37.0%
2018	86.0%	4.0%	48.3%
2019	84.0%	-2.0%	52.1%
2020	84.0%	0.0%	61.8%

Outcomes from 2020 only up to March 17, 2020.

© 2025 Enns B et al. *JAMA Network Open*.

eTable 4. Priors and posteriors for calibrated parameters

Parameter	Prior<br>Mean (95% CI)	Posterior<br>(initiator)<br>Mean (95% CI)	Posterior<br>(per-protocol)<br>Mean (95% CI)
Non-fent overdose rate (OAT)	0.0013 (0.001, 0.0016)	0.0012 (0.0011, 0.0012)	0.001 (0.0009, 0.0011)
Fentanyl overdose mult (prevalence)	1 (0, 5)	1.6958 (1.6093, 1.7787)	1.7156 (1.6186, 1.8152)
Fentanyl overdose mult (delta)	1 (0, 5)	0.6881 (0.4879, 0.8895)	0.6563 (0.4297, 0.8759)
Fatal overdose rate (OAT)	0.0242 (0.0147, 0.0472)	0.0071 (0.0066, 0.0076)	0.007 (0.0063, 0.0078)
Non-overdose mortality mult (OAT)	4.9926 (3.2958, 8.3786)	4.1125 (3.9755, 4.2472)	3.9246 (3.7828, 4.0613)
Remain out-of-treatment (scale)	0.025 (0, 0.05)	0.0456 (0.0373, 0.0556)	0.0156 (0.0085, 0.0235)
Remain out-of-treatment (shape)	1 (0, 2)	0.9736 (0.9018, 1.0393)	1.5999 (1.4226, 1.8166)
Remain long-term abstinence (scale)	0.0215 (0.0325, 0.0135)	0.0278 (0.026, 0.031)	0.0156 (0.013, 0.0183)
Remain long-term abstinence (shape)	1 (0, 2)	0.9827 (0.9335, 1.034)	1.3528 (1.2646, 1.4529)

CI: credible interval; OAT: opioid agonist therapy; Mult: multiplier.

eTable 5. Unique individuals initiating OAT episodes in each year

Year	Incident OAT clients		Experienced OAT clients	
	Methadone	BNX	Methadone	BNX
2010	1943	245	2215	156
2011	2771	432	3429	367
2012	3144	621	4391	601
2013	2888	679	4746	791
2014	2895	725	5510	1056
2015	2930	843	5432	1364
2016	2890	1779	5772	2159
2017	2474	2669	6169	3053
2018	2042	2765	6561	3555
2019	1756	2584	7028	3848
2020	651	954	3863	1922

Outcomes from 2020 only up to March 17, 2020; some individuals may initiate episodes as incident and experienced OAT clients in the same year; OAT: opioid agonist treatment; BNX: buprenorphine/naloxone.

© 2025 Enns B et al. *JAMA Network Open*.

eTable 6. Incremental life years from alternative treatment policies of exclusively buprenorphine/naloxone versus exclusively methadone

Time horizon	Incremental life years gained (buprenorphine/naloxone vs. methadone)		
	Initiator (primary analysis)	Per-protocol (sensitivity analysis)	High-dose (sensitivity analysis)
2010	-3 (-9, 0)	-3 (-8, 0)	-1 (-8, 2)
2010 - 2012	-60 (-137, -10)	-49 (-123, -1)	-26 (-119, 22)
2010 - 2014	-216 (-470, -52)	-171 (-419, -13)	-96 (-409, 63)
2010 - 2016	-540 (-1139, -160)	-419 (-1019, -44)	-250 (-997, 133)
2010 - 2018	-1163 (-2378, -387)	-893 (-2157, -115)	-556 (-2116, 243)
2010 - 2020	-1602 (-3249, -549)	-1229 (-2959, -168)	-776 (-2912, 319)

© 2025 Enns B et al. *JAMA Network Open*.

eTable 7. Percentage of simulations with incremental life years below threshold, 2010 – 2020

Incremental life year threshold	Percentage of simulations less than threshold (buprenorphine/naloxone vs. methadone)		
	Initiator (primary analysis)	Per-protocol (sensitivity analysis)	High-dose (sensitivity analysis)
0	100.0%	99.4%	85.2%
-500	98.2%	87.4%	55.5%
-1000	81.8%	57.7%	30.8%
-1500	50.3%	29.6%	16.6%
-2000	24.1%	13.1%	8.4%
-2500	9.9%	5.2%	4.4%
-3000	4.0%	2.3%	2.3%

© 2025 Enns B et al. *JAMA Network Open*.

eTable 8. Incremental fatal overdoses from alternative treatment policies of exclusively buprenorphine/naloxone versus exclusively methadone

Time horizon	Incremental fatal overdoses (buprenorphine/naloxone vs. methadone)		
	Initiator (primary analysis)	Per-protocol (sensitivity analysis)	High-dose (sensitivity analysis)
2010	4 (2, 9)	4 (1, 9)	2 (-1, 9)
2010 - 2012	22 (11, 40)	18 (4, 40)	12 (-2, 39)
2010 - 2014	56 (29, 96)	43 (11, 96)	30 (-3, 96)
2010 - 2016	116 (62, 197)	87 (22, 197)	64 (-5, 201)
2010 - 2018	189 (102, 321)	145 (36, 325)	106 (-8, 332)
2010 - 2020	221 (119, 376)	170 (42, 382)	126 (-9, 391)

© 2025 Enns B et al. *JAMA Network Open*.

eTable 9. Incremental all-cause deaths from alternative treatment policies of exclusively buprenorphine/naloxone versus exclusively methadone

Time horizon	Incremental all-cause deaths (buprenorphine/naloxone vs. methadone)		
	Initiator (primary analysis)	Per-protocol (sensitivity analysis)	High-dose (sensitivity analysis)
2010	7 (1, 18)	6 (0, 16)	3 (-3, 15)
2010 - 2012	38 (9, 81)	30 (3, 72)	17 (-11, 70)
2010 - 2014	87 (28, 178)	66 (8, 159)	41 (-19, 156)
2010 - 2016	166 (63, 326)	125 (20, 296)	81 (-28, 295)
2010 - 2018	260 (103, 502)	197 (34, 462)	132 (-40, 465)
2010 - 2020	303 (120, 589)	231 (39, 542)	156 (-46, 549)

© 2025 Enns B et al. *JAMA Network Open*.

eFigure 1. Diagram of model health states and possible transitions

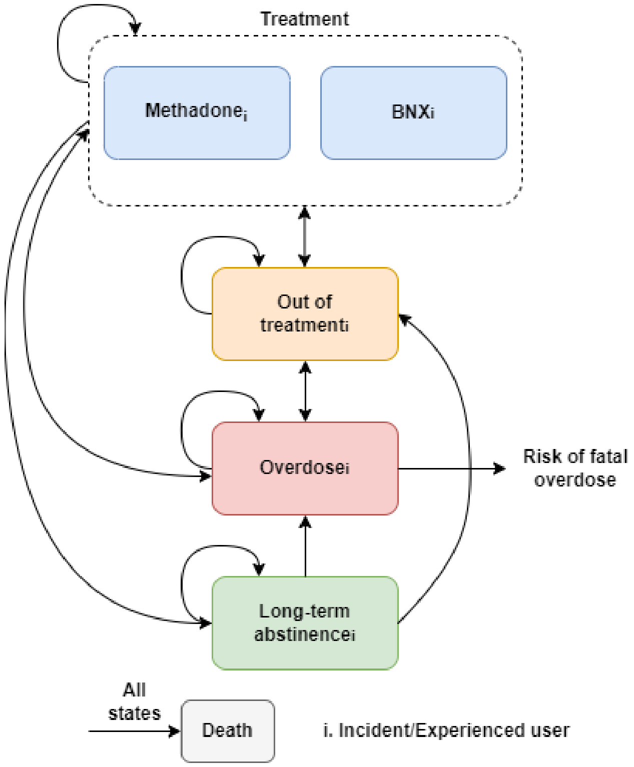

BNX: buprenorphine/naloxone.

© 2025 Enns B et al. *JAMA Network Open*.

eFigure 2. Comparison of fitted model status quo outputs with calibration targets (initiator analysis)

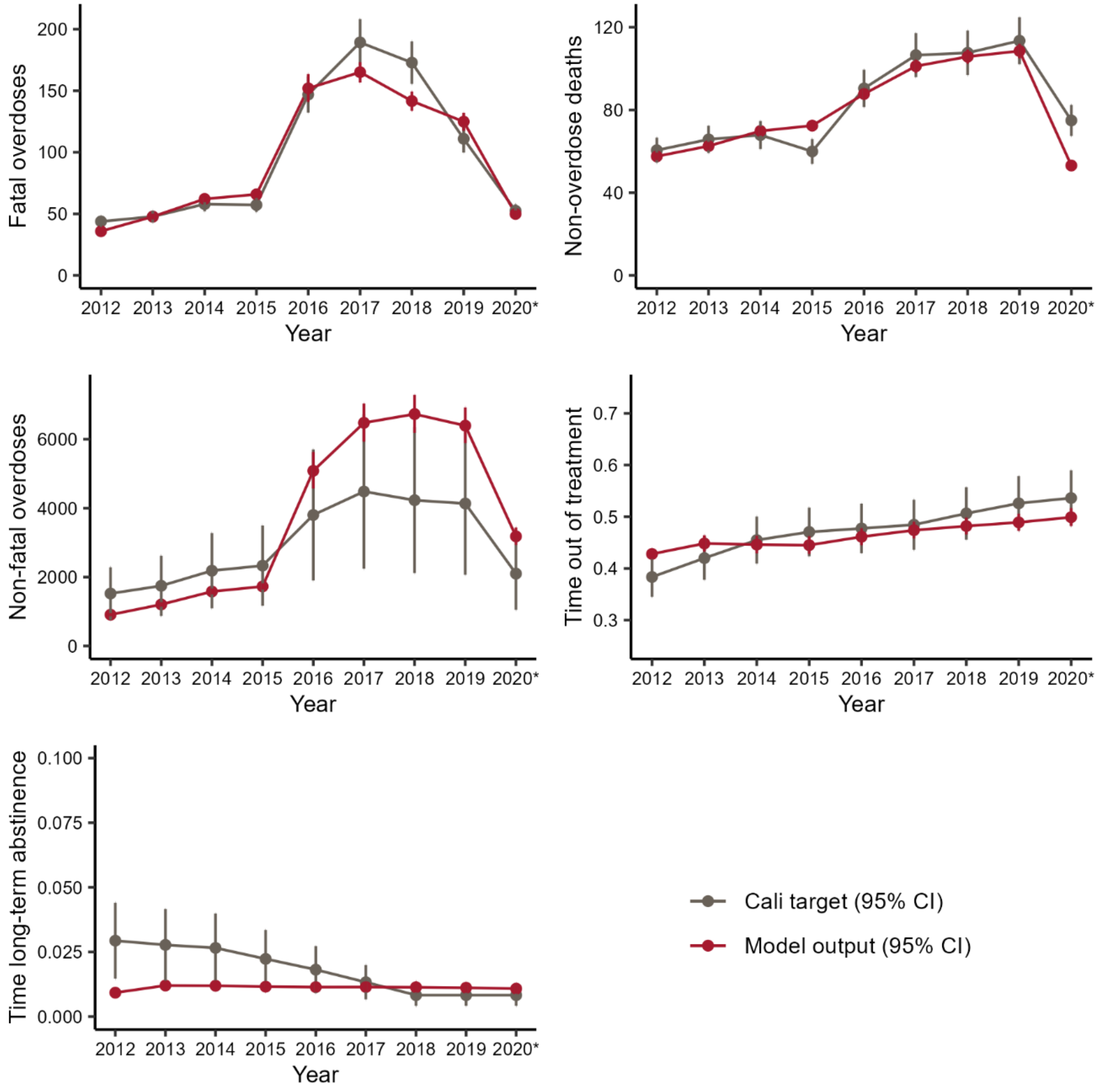

*Outcomes from 2020 only up to March 17, 2020.

© 2025 Enns B et al. *JAMA Network Open*.

eFigure 3. Comparison of fitted model status quo outputs with calibration targets (per-protocol analysis)

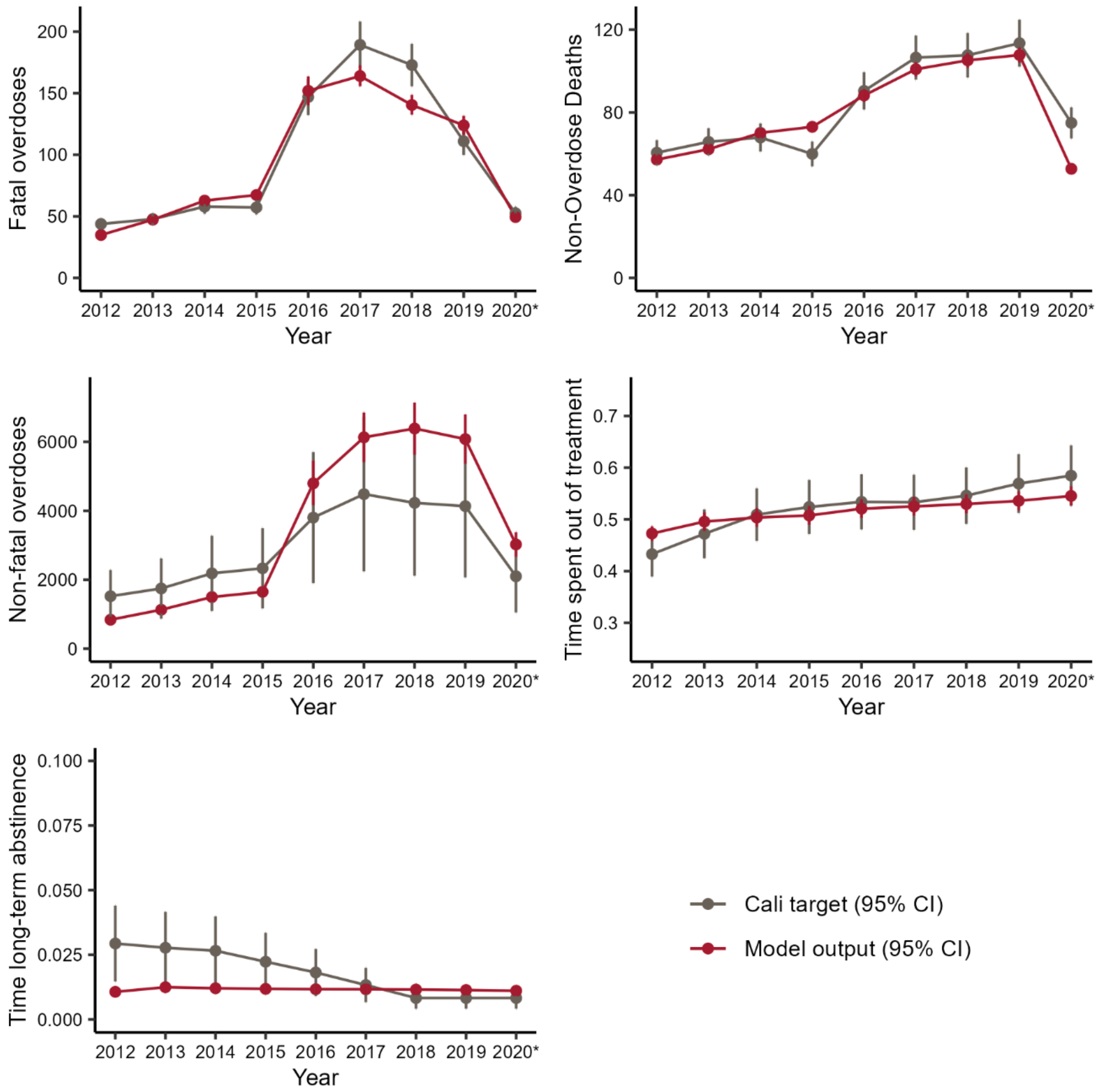

*Outcomes from 2020 only up to March 17, 2020.

© 2025 Enns B et al. *JAMA Network Open*.

eFigure 4. Prior and posterior distributions for calibrated model parameters (initiator analysis)

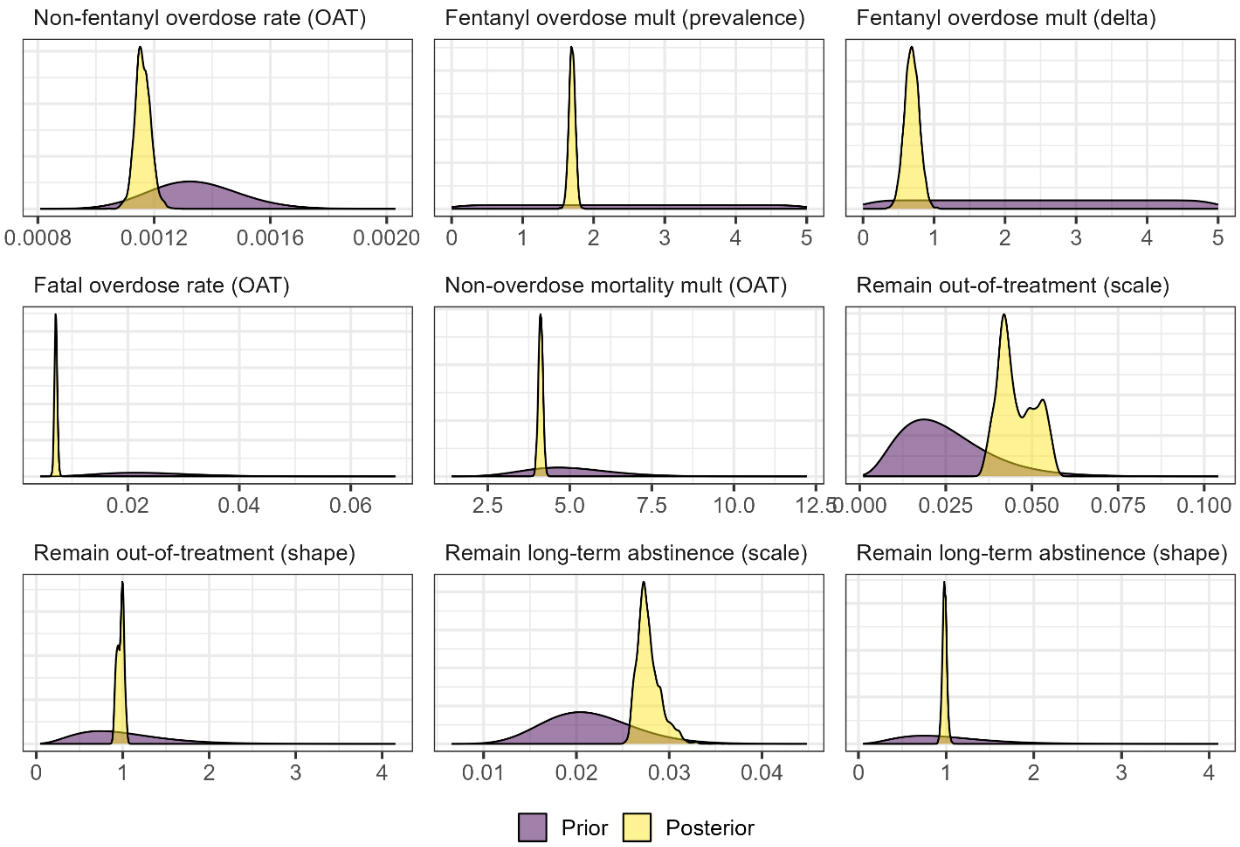

OAT: opioid agonist treatment; Mult: multiplier of base rate.

© 2025 Enns B et al. *JAMA Network Open*.

eFigure 5. Posterior distributions and correlations for calibrated model parameters (initiator analysis)

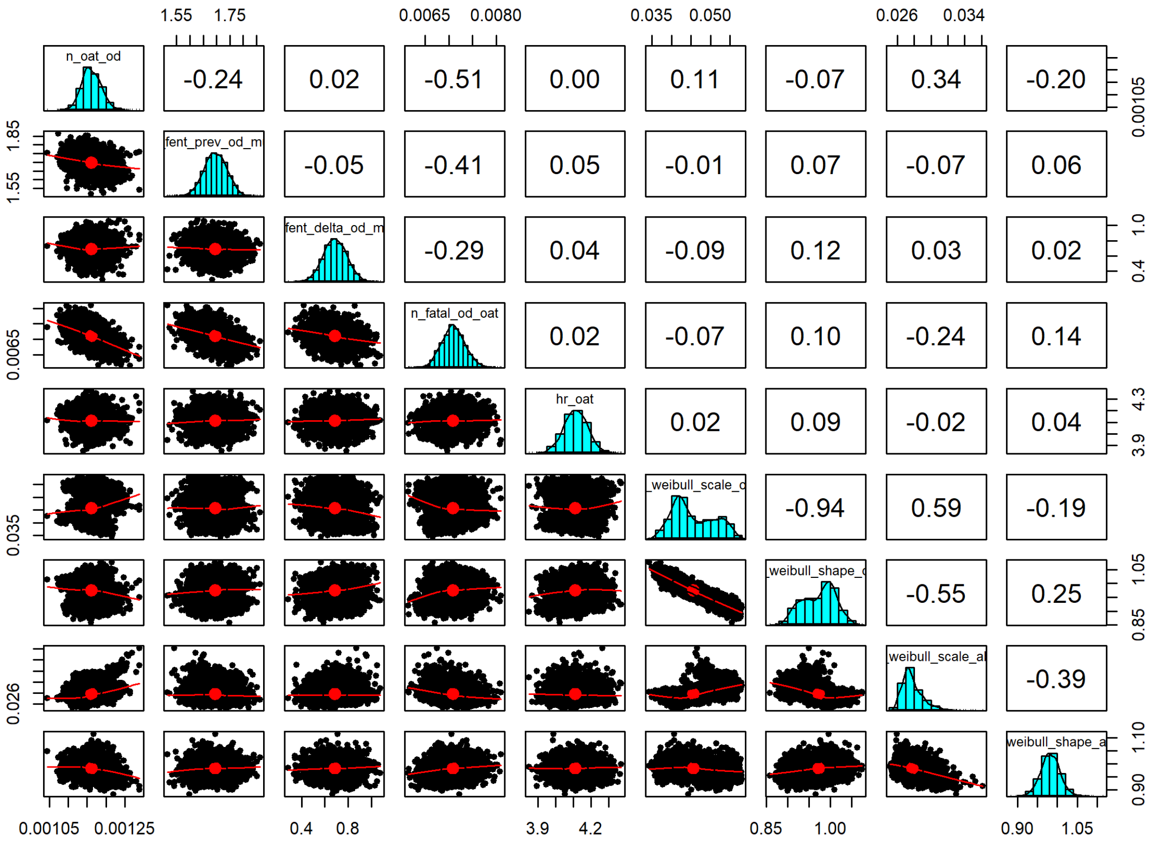

n_oat_od: Baseline overdose rate in treatment (all OAT) with no fentanyl exposure, second week+; fent_prev_od_mult: Multiplier of overdose risk tied to fentanyl prevalence; fent_delta_od_mult: Multiplier of overdose risk tied to yearly change in fentanyl prevalence; n_fatal_od_oat: Baseline fatal overdose rate in OAT, conditional on overdose; hr_oat: Multiplier of non-overdose mortality for OAT relative to general population.

© 2025 Enns B et al. *JAMA Network Open*.

eFigure 6. Prior and posterior distributions for calibrated model parameters (per-protocol analysis)

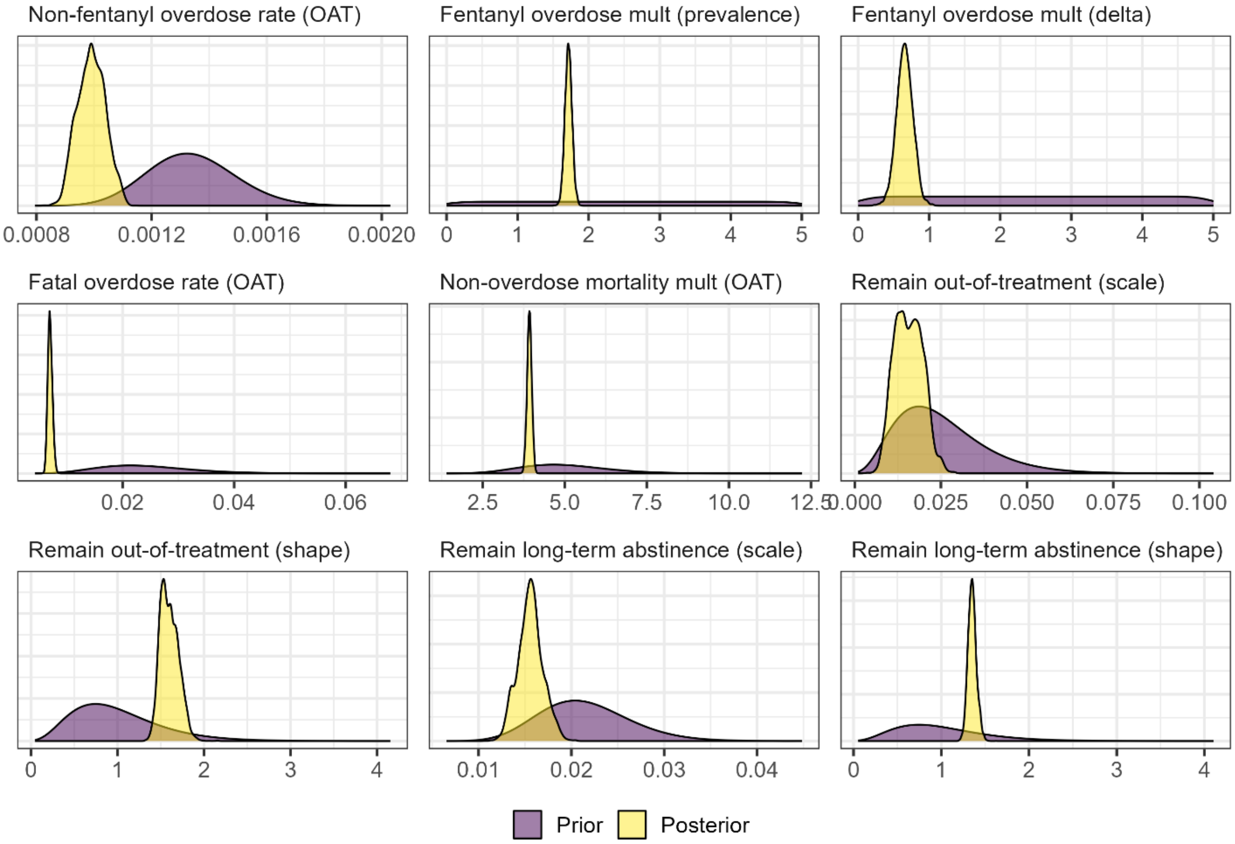

OAT: Opioid agonist treatment; Mult: Multiplier of baseline rate.

© 2025 Enns B et al. *JAMA Network Open*.

eFigure 7. Posterior distributions and correlations for calibrated model parameters (per-protocol analysis)

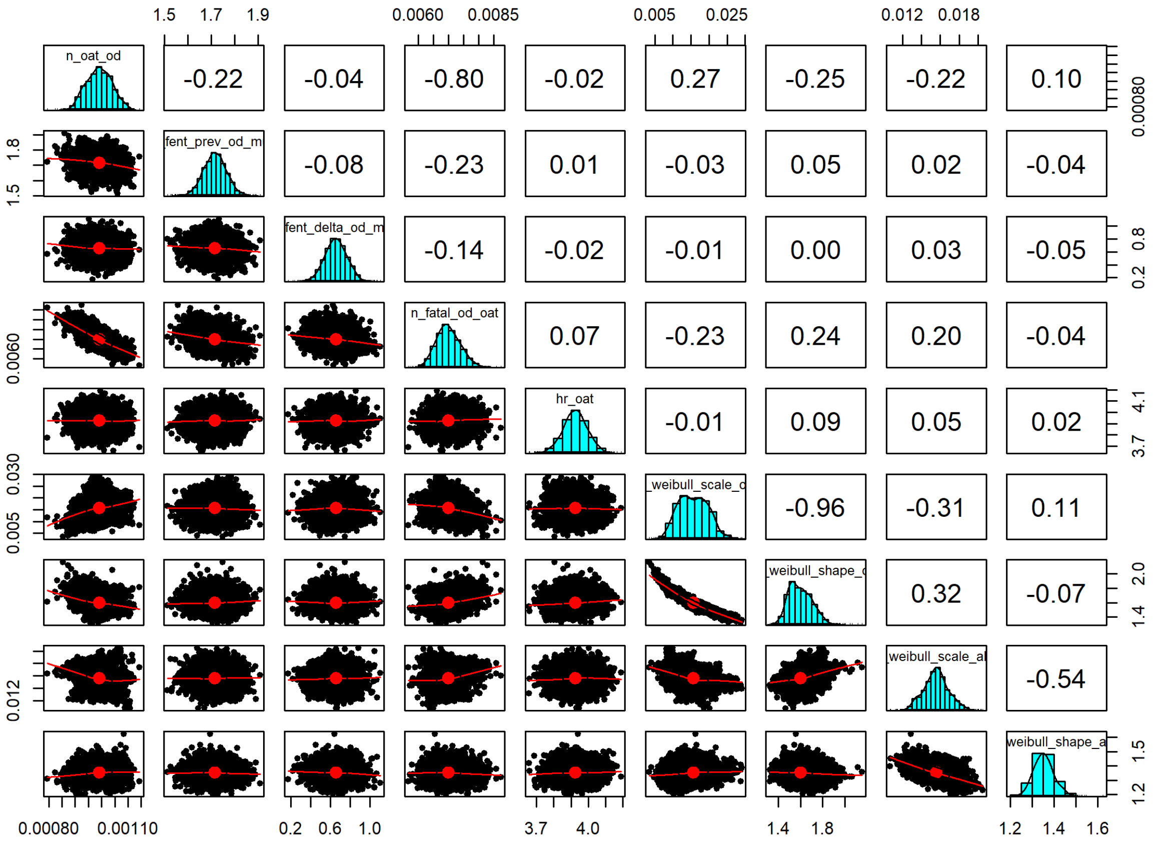

n_oat_od: Baseline overdose rate in treatment (all OAT) with no fentanyl exposure, second week+; fent_prev_od_mult: Multiplier of overdose risk tied to fentanyl prevalence; fent_delta_od_mult: Multiplier of overdose risk tied to yearly change in fentanyl prevalence; n_fatal_od_oat: Baseline fatal overdose rate in OAT, conditional on overdose; hr_oat: Multiplier of non-overdose mortality for OAT relative to general population.

© 2025 Enns B et al. *JAMA Network Open*.

eFigure 8. Plot of model-projected incident and experienced user population strata over time compared to observed data

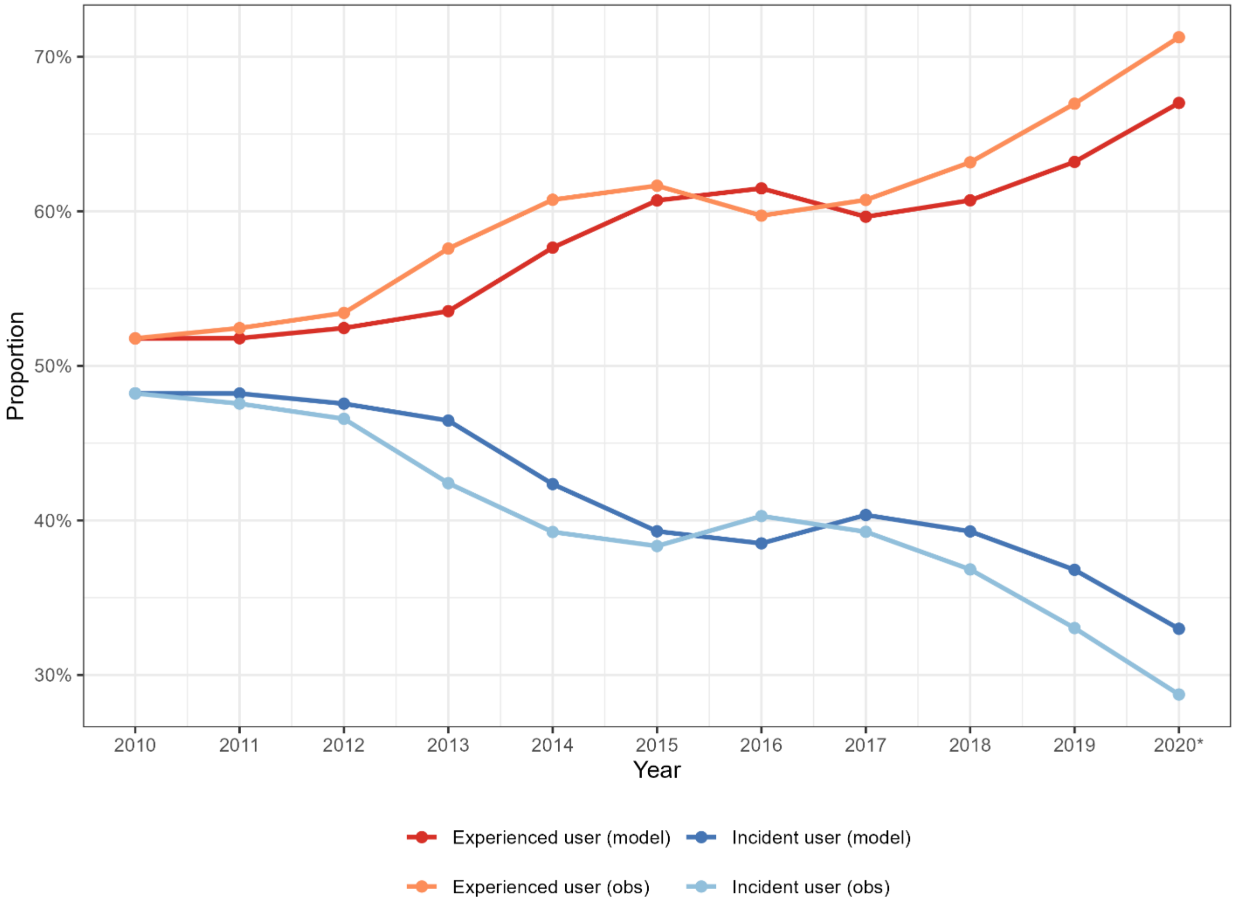

© 2025 Enns B et al. *JAMA Network Open*.

eFigure 9. Total cumulative life-years projected for each alternative treatment policy (primary analysis)

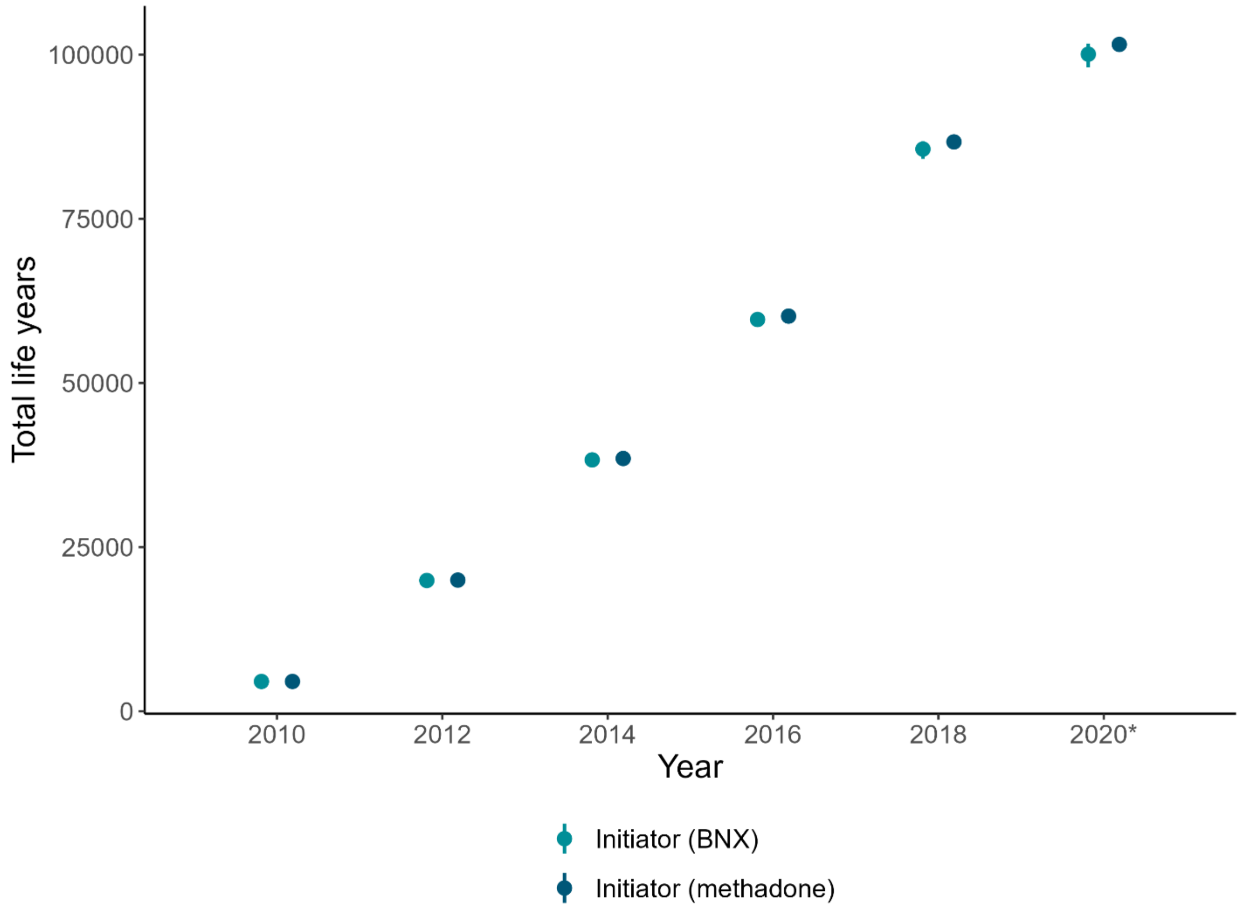

*Outcomes from 2020 only up to March 17, 2020.

© 2025 Enns B et al. *JAMA Network Open*.

eFigure 10: Model trace for buprenorphine/naloxone (initiator analysis)

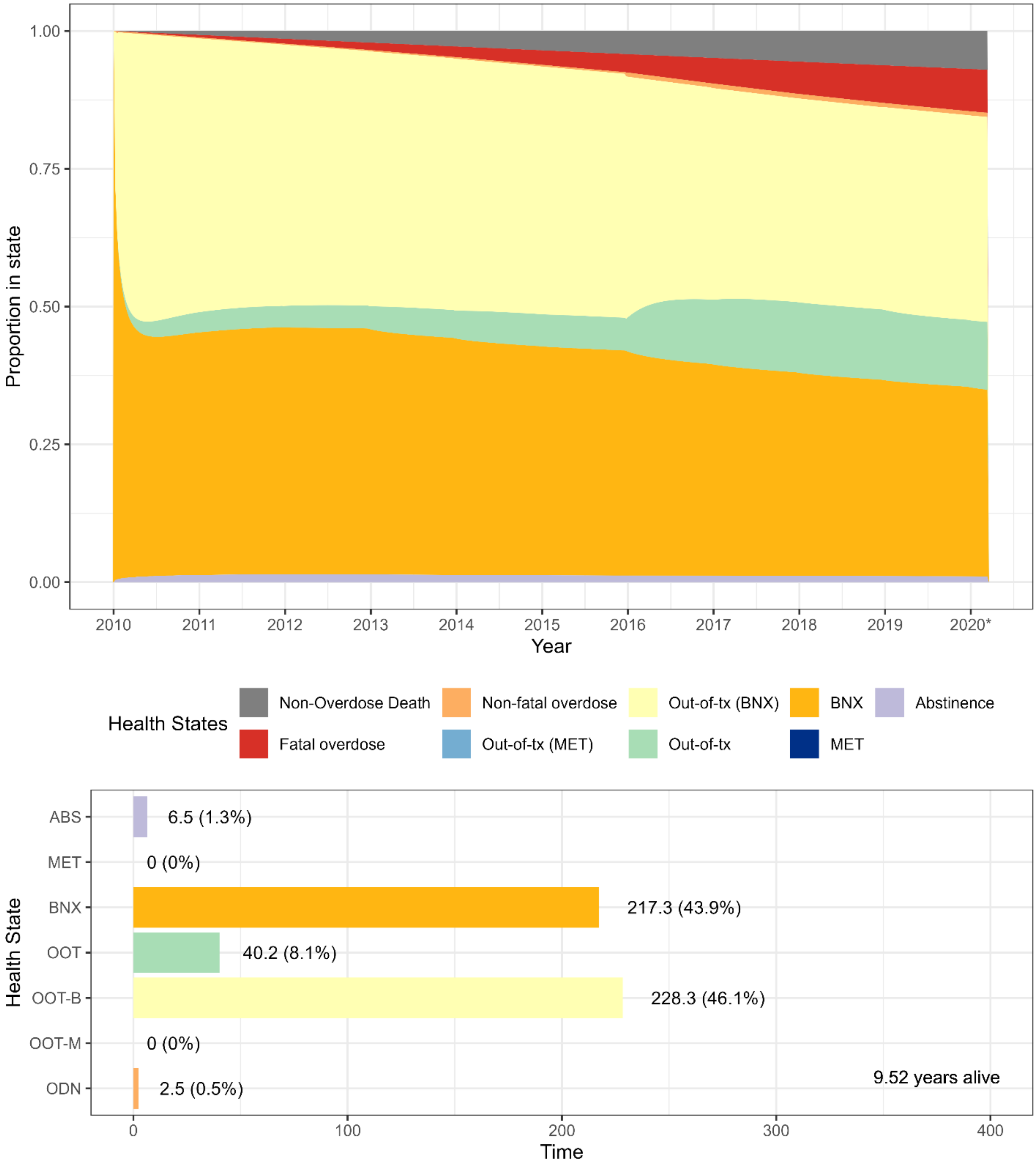

*Outcomes from 2020 only up to March 17, 2020; ABS: long-term abstinence; MET: methadone; BNX: buprenorphine/naloxone; OOT: opioid use following out-of-treatment health state (long-term abstinence or non-fatal overdose); OOT-B: opioid use following discontinuation from buprenorphine/naloxone; OOT-M: opioid use following discontinuation from methadone; ODN: non-fatal overdose.

© 2025 Enns B et al. *JAMA Network Open*.

eFigure 11: Model trace for methadone (initiator analysis)

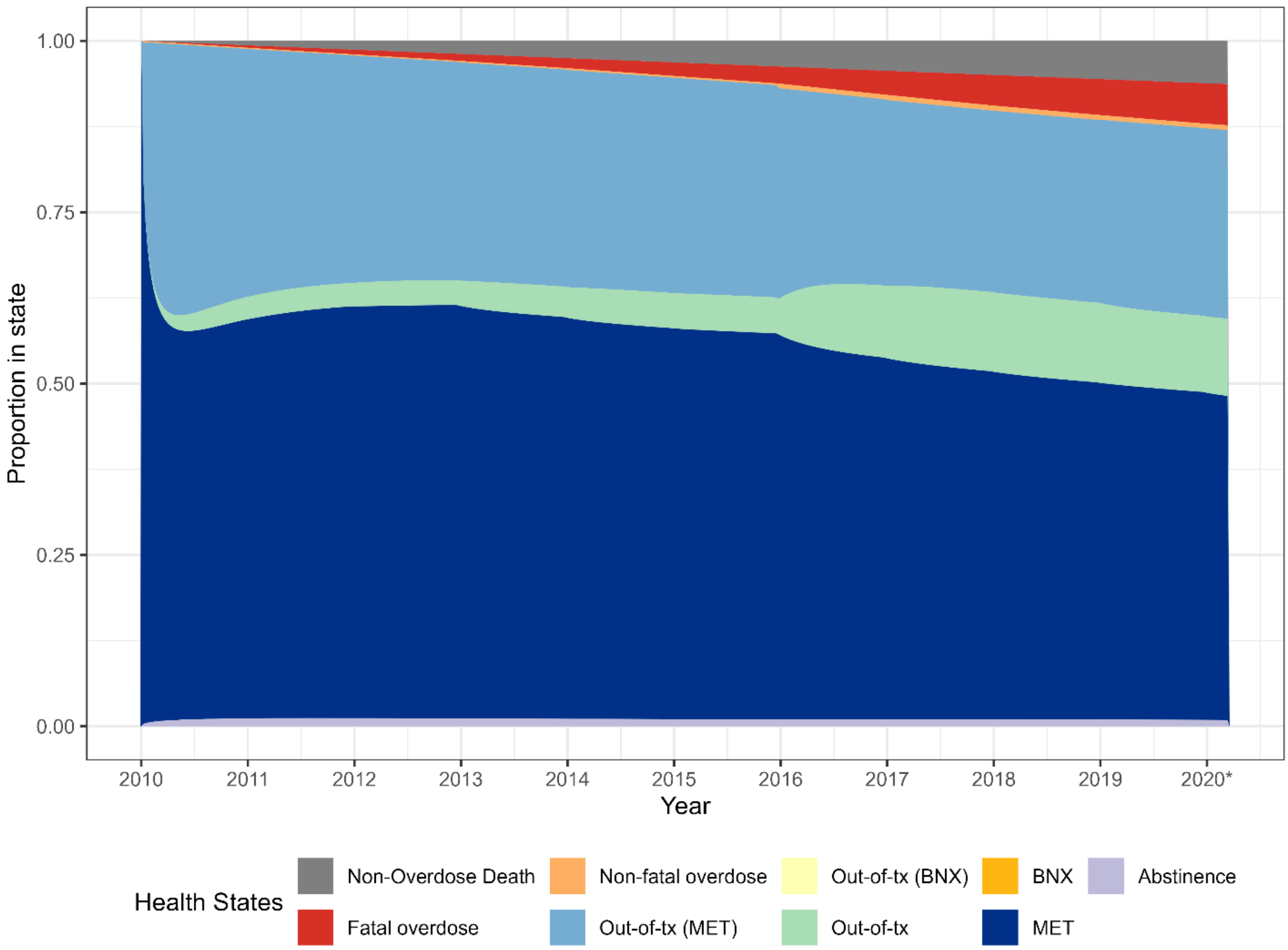

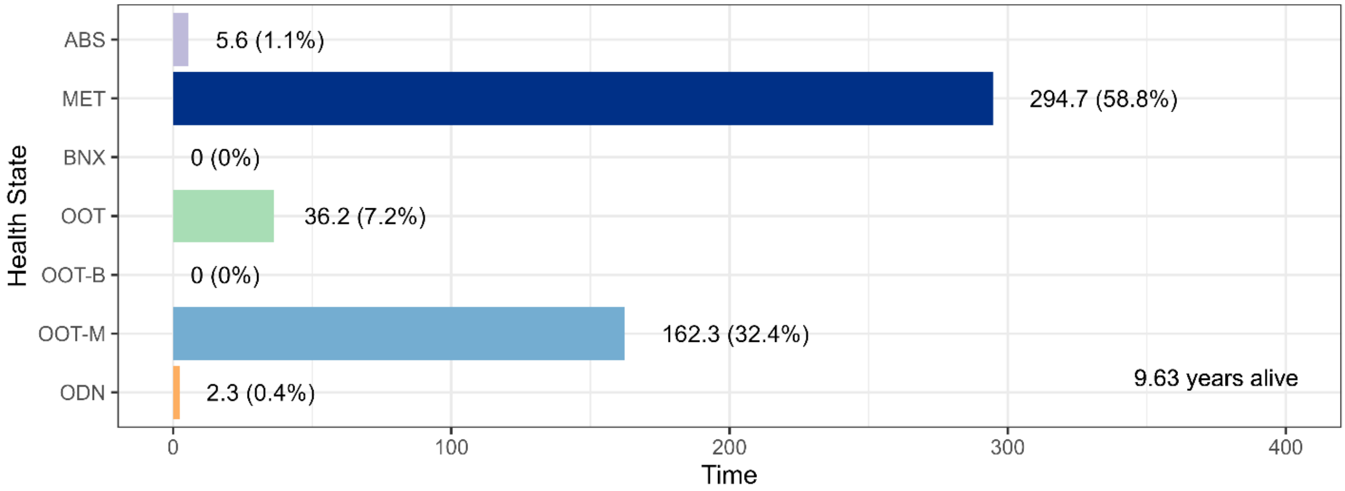

*Outcomes from 2020 only up to March 17, 2020; ABS: long-term abstinence; MET: methadone; BNX: buprenorphine/naloxone; OOT: opioid use following out-of-treatment health state (long-term abstinence or non-fatal overdose); OOT-B: opioid use following discontinuation from buprenorphine/naloxone; OOT-M: opioid use following discontinuation from methadone; ODN: non-fatal overdose.

© 2025 Enns B et al. *JAMA Network Open*.

eFigure 12: Model trace for buprenorphine/naloxone (per-protocol analysis)

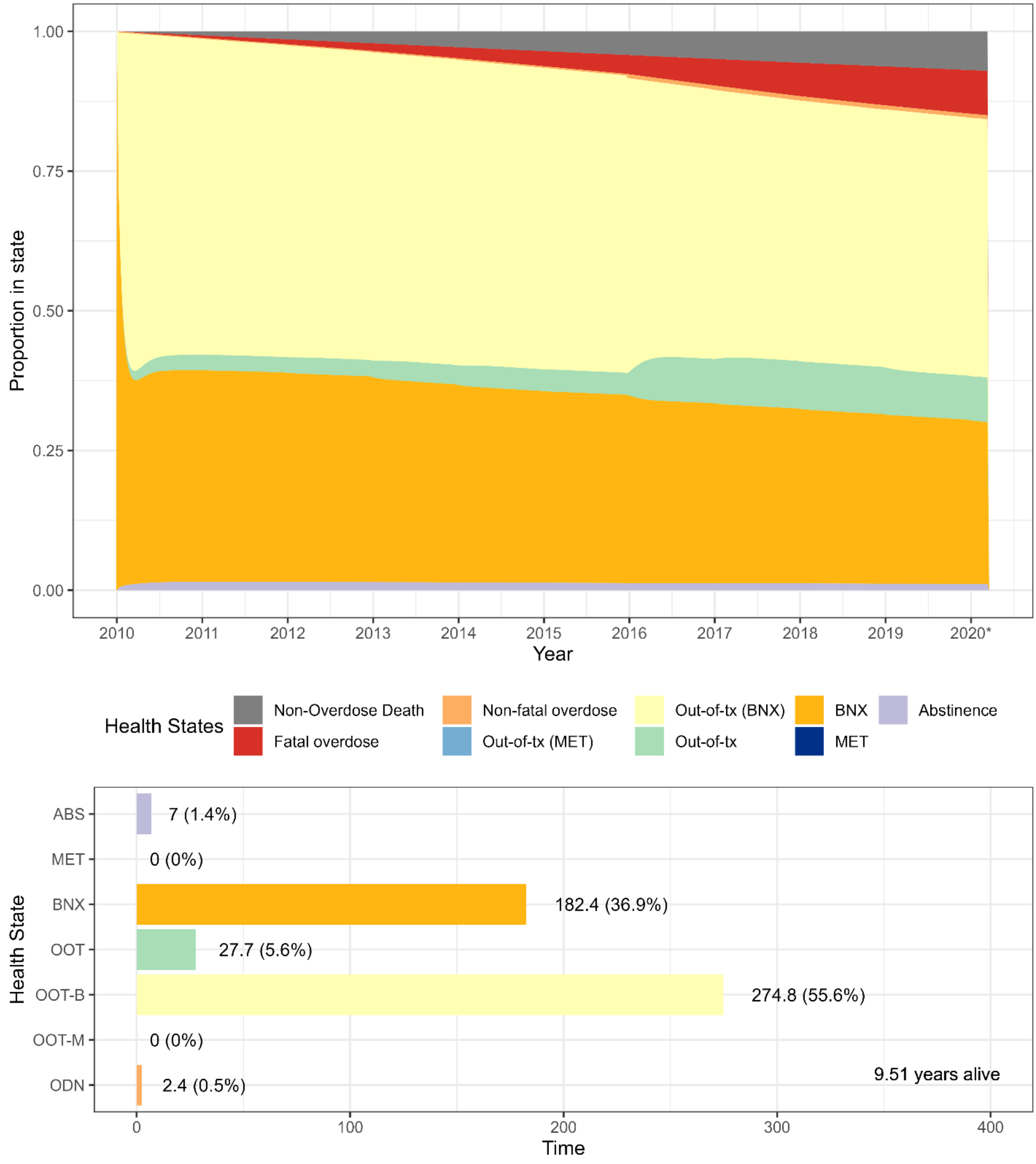

*Outcomes from 2020 only up to March 17, 2020; ABS: long-term abstinence; MET: methadone; BNX: buprenorphine/naloxone; OOT: opioid use following out-of-treatment health state (long-term abstinence or non-fatal overdose); OOT-B: opioid use following discontinuation from buprenorphine/naloxone; OOT-M: opioid use following discontinuation from methadone; ODN: non-fatal overdose.

© 2025 Enns B et al. *JAMA Network Open*.

eFigure 13: Model trace for methadone (per-protocol analysis)

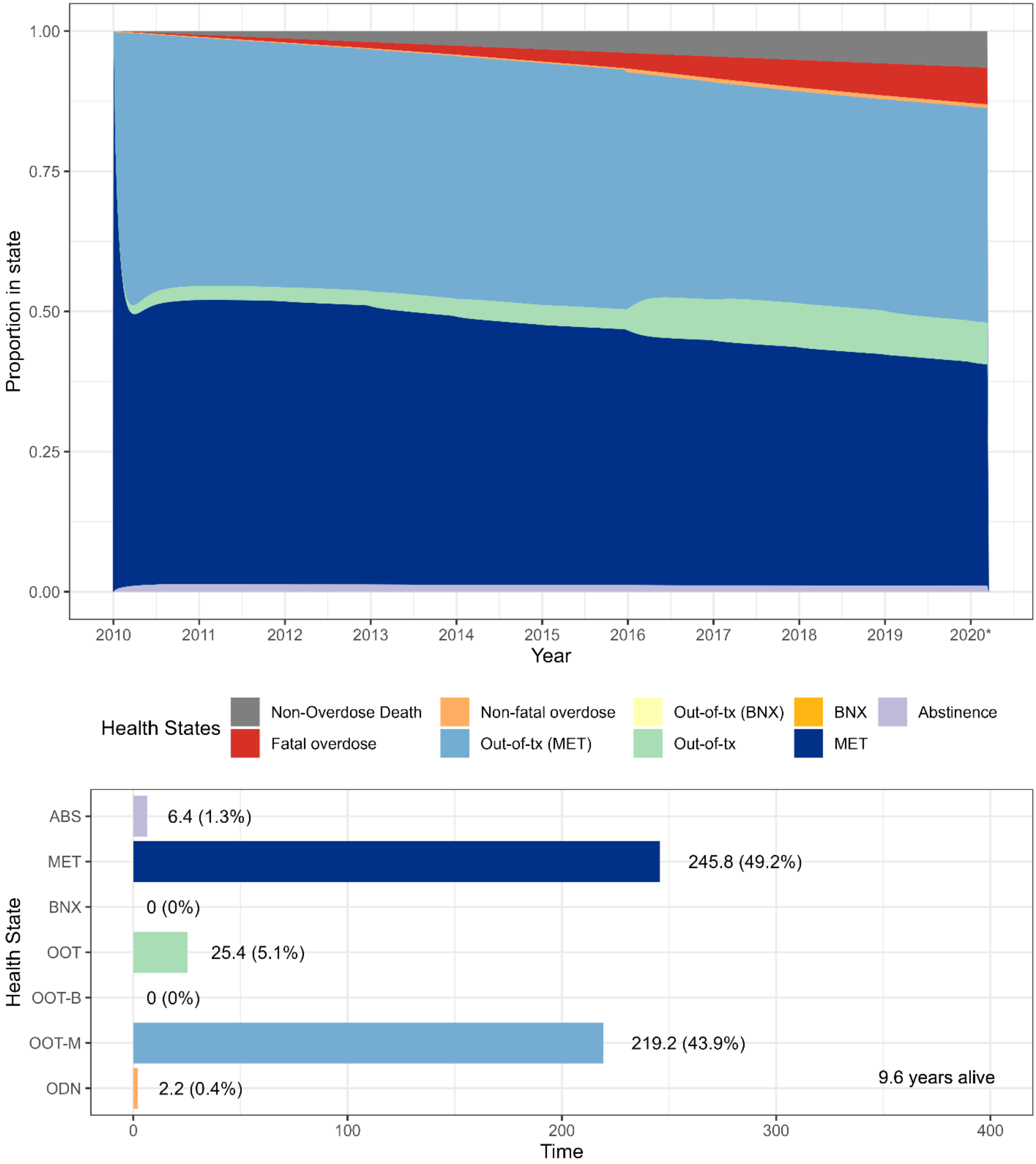

*Outcomes from 2020 only up to March 17, 2020; ABS: long-term abstinence; MET: methadone; BNX: buprenorphine/naloxone; OOT: opioid use following out-of-treatment health state (long-term abstinence or non-fatal overdose); OOT-B: opioid use following discontinuation from buprenorphine/naloxone; OOT-M: opioid use following discontinuation from methadone; ODN: non-fatal overdose.

© 2025 Enns B et al. *JAMA Network Open*.

eFigure 14. Cumulative incremental life-years for alternative buprenorphine/naloxone versus methadone treatment policies stratified by all-incident and all-experienced OAT clients

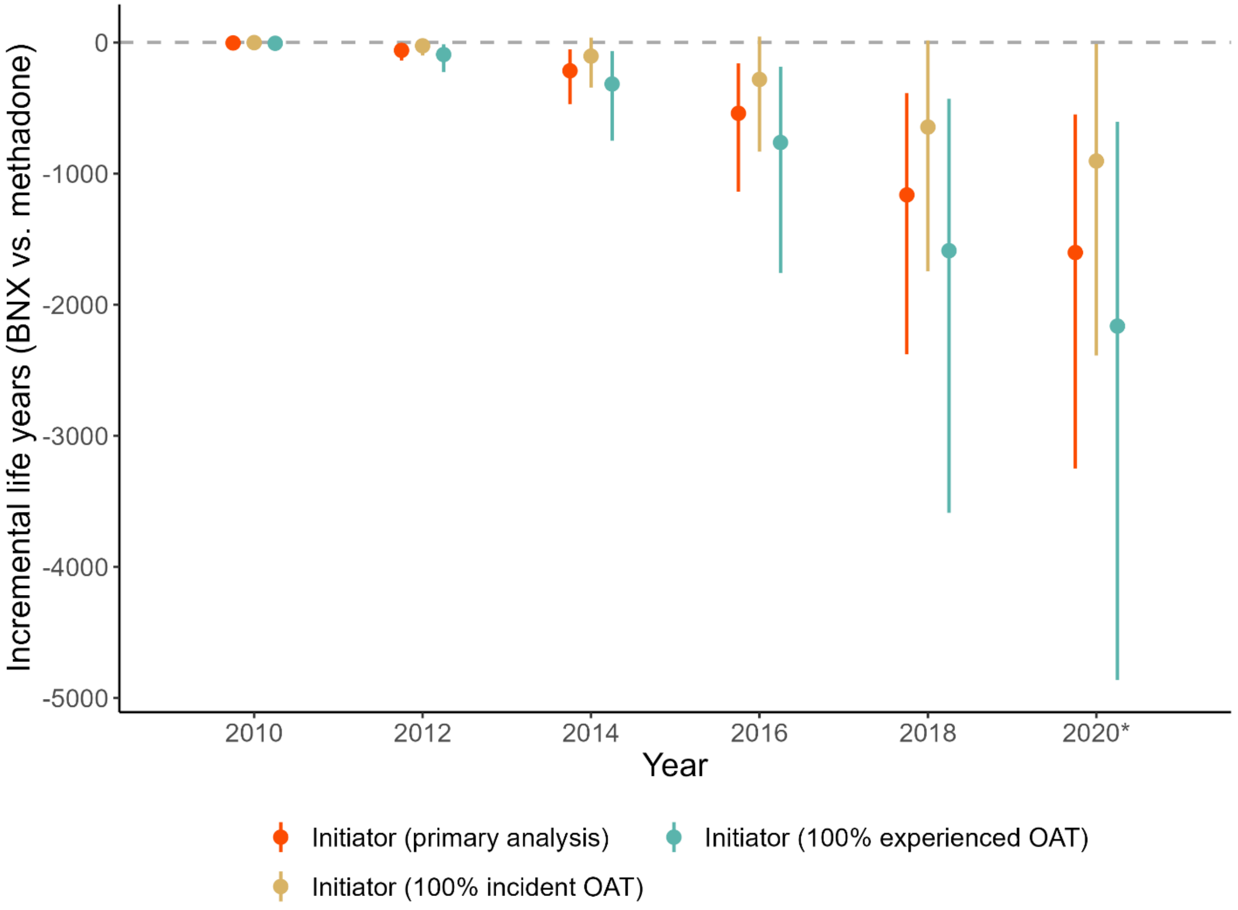

*Outcomes from 2020 only up to March 17, 2020; OAT: opioid agonist treatment; BNX: buprenorphine/naloxone.

© 2025 Enns B et al. *JAMA Network Open*.

eFigure 15. Univariate sensitivity analysis on life-years gained buprenorphine/naloxone versus methadone (initiator analysis)

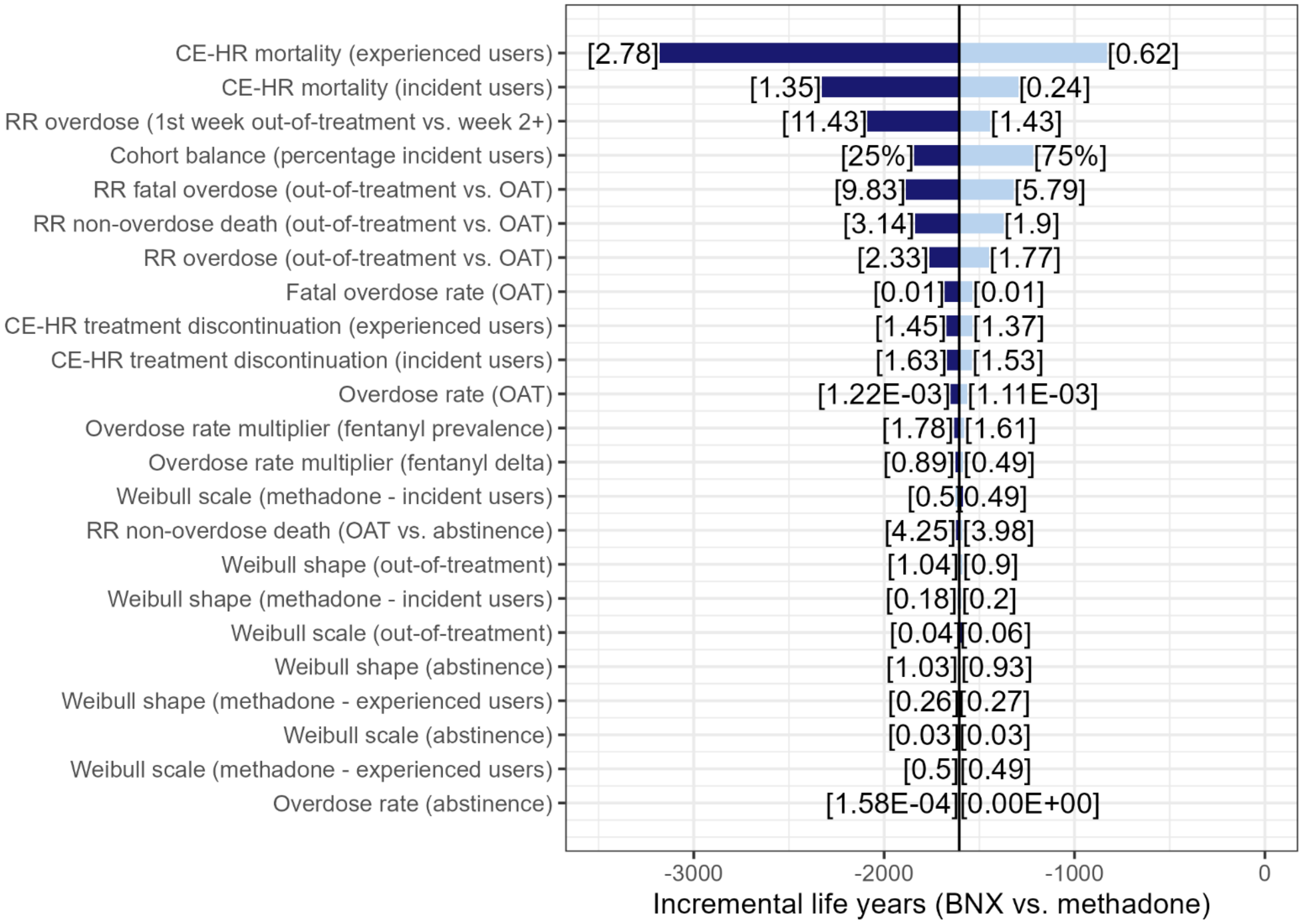

HR: hazard ratio; RR: rate ratio; OAT: opioid agonist treatment.

© 2025 Enns B et al. *JAMA Network Open*.
