# Supplementary material for: Population-Level Health Benefits and Harms Associated With Buprenorphine/Naloxone vs Methadone
Source: JAMA Netw Open. 2025 Dec 26;8(12):e2551337. doi: 10.1001/jamanetworkopen.2025.51337 (PMC12743284; doi:10.1001/jamanetworkopen.2025.51337)
Supplement: Supplement 2. — Data Sharing Statement [file jamanetwopen-e2551337-s002.pdf]

Data Sharing Statement

Enns. Population-Level Health Benefits and Harms Associated With Buprenorphine/Naloxone vs Methadone. *JAMA Netw Open*. Published online December 26, 2025. doi:10.1001/jamanetworkopen.2025.51337

Data
Data available: No

Additional Information
Explanation for why data not available: We were unable to share the individual-level administrative data set due to privacy restrictions, however, all code and inputs necessary to run the model and generate the results from this study are available at: https://github.com/HERU-modeling/BNX-MET-CE
